# Bark presence shifts fungal guild composition with varying effects on fungal diversity and biomass in a tropical forest

**DOI:** 10.1093/ismeco/ycag258

**Published:** 2026-09-06

**Authors:** Jean Evans Israel Codjia, Busayo Joshua Babalola, Weiming Hu, Yun-Qiang Yang, Sewanou H Honfo, Ekananda Paudel, Douglas Schaefer, Kun-Fang Cao, Jian-Chu Xu, Yong-Ping Yang, Rhett D Harrison, Gbadamassi G O Dossa

**Affiliations:** Yunnan Key Laboratory of Forest Ecosystem Stability and Global Change, Xishuangbanna Tropical Botanical Garden, Chinese Academy of Sciences, Menglun, Mengla, Yunnan 666303, China; Yunnan International Joint Laboratory for the Conservation and Utilization of Tropical Timber Tree Species, Xishuangbanna Tropical Botanical Garden, Chinese Academy of Sciences, Mengla, Yunnan 666303, China; Research Unit Tropical Mycology and Plant-Soil Fungi Interactions, Faculty of Agronomy, University of Parakou, Parakou BP 123, Benin; Department of Plant Biology and Plant Pathology, University of Georgia, Athens, GA 30602, United States; Division of Natural Sciences, Oglethorpe University, Atlanta, GA, United States; Division of Gastroenterology, Hepatology and Nutrition, Children’s Hospital of Philadelphia, Philadelphia, PA 19104, United States; Yunnan International Joint Laboratory for the Conservation and Utilization of Tropical Timber Tree Species, Xishuangbanna Tropical Botanical Garden, Chinese Academy of Sciences, Mengla, Yunnan 666303, China; Division of Statistics, Montreal Health Innovations Coordinating Center (MHICC), Montreal Heart Institute, Université de Montréal, Montreal, Canada; Nepal Academy of Science and Technology, Khumaltar, Lalitpur, Nepal; Centre for Mountain Futures, Kunming Institute of Botany, Chinese Academy of Science, Kunming, Yunnan 650201, China; Ecophysiology and Evolution Group, Guangxi Key Laboratory of Forest Ecology and Conservation, and College of Forestry, Guangxi University, Nanning, China; Centre for Mountain Futures, Kunming Institute of Botany, Chinese Academy of Science, Kunming, Yunnan 650201, China; Key Laboratory of Economic Plants and Biotechnology, Kunming Institute of Botany, Chinese Academy of Sciences, Kunming 650201, China; Yunnan International Joint Laboratory for the Conservation and Utilization of Tropical Timber Tree Species, Xishuangbanna Tropical Botanical Garden, Chinese Academy of Sciences, Mengla, Yunnan 666303, China; Landscape Alliance, Lusaka, Zambia; Yunnan Key Laboratory of Forest Ecosystem Stability and Global Change, Xishuangbanna Tropical Botanical Garden, Chinese Academy of Sciences, Menglun, Mengla, Yunnan 666303, China

**Keywords:** alpha diversity, beta diversity, ecosystem function, phospholipid fatty acids, assembly processes

## Abstract

Bark influences wood decomposition, but the mechanisms are unclear. We investigated fungal community composition, microbial biomass, and wood physicochemical condition during a wood decomposition experiment. We carried out a 2-year common garden experiment in a tropical seasonal rainforest, China, with logs from five tree species, comparing intact-bark versus bark-removed treatments over 0, 18, and 24 months of field exposure. We sequenced wood-inhabiting fungi, measured microbial biomass, and assessed wood chemical properties over time. We anticipated bark presence would alter fungal community composition and increase diversity, decay types, and microbial biomass. Wood with bark-removed had a higher relative abundance of Ascomycota, whereas bark presence fostered Basidiomycota. Soft rot fungi showed the highest relative abundance, followed by white rot fungi and brown rot fungi. However, bark presence was associated with higher relative abundance of white rot fungi, during early decay. Wood chemical properties and moisture (water content) availability, were correlated with the dominance of saprotrophs, rather than bark status. Despite compositional shifts, bark status did not influence fungal alpha diversity at the bark effects level. Bark status also did not directly influence fungal and bacterial biomass. Microbial biomass dynamics were instead linked to changes in wood physicochemical properties as decomposition progressed. Fungal community turnover dominated over time in bark-removed treatments, while bark presence redirected community assembly toward nestedness at the advanced decay stages. Overall, bark presence was linked with a shift toward more deterministic, habitat-filtered microbial community assembly, suggesting a multi-guild, multi-factor framework to predict bark effects on wood decay in forests.

## Introduction

Deadwood decomposition is a fundamental process in forest ecosystems, regulating the turnover of carbon (C) and nutrients and influencing biodiversity as well as ecosystem function [1–3]. Globally, deadwood stores an estimated 73 ± 6 petagrams (Pg) of C, roughly 8% of total forest carbon, and releases CO₂ at rates similar to fossil fuel combustion [4, 5] with an estimation of 10.9 (±3.2) Pg of C released annually [6]. Understanding the mechanisms that control decomposition is therefore critical for modeling terrestrial carbon cycling under climate change [3, 5]. Beyond its role in biogeochemical fluxes, decaying wood provides habitat for a diverse assemblage of fungi, bacteria, and invertebrates [3, 7]. These communities are shaped by both substrate traits and microclimatic conditions, making wood decomposition a microecological laboratory for studying how biotic and abiotic factors interact to regulate ecosystem processes [8, 9].

Beyond its role as a passive barrier, bark is a chemically complex and ecologically active interface between living and dead plant tissues and comprises up to 20% of the above-ground tree biomass [10, 11]. Structurally, bark consists of outer protective periderm and inner phloem tissues enriched in suberin, lignin, and phenolic compounds [11, 12]. These compounds confer resistance to desiccation, microbial attack, and herbivory, but they also influence postmortem decay dynamics by modifying the physicochemical microenvironment of the underlying xylem [10, 11]. Bark typically exhibits higher nitrogen, phosphorus, and mineral content than xylem [11, 13], yet its hydrophobic components slow oxygen and water diffusion [14]. As a result, bark can act as an ecological filter that influences which decomposers colonize xylem and how they function [15, 16]. For instance, bark retains moisture and moderates temperature fluctuations [10, 14], potentially enhancing microbial activity in coarse woody debris (5–9 cm in diameter) and subsequently accelerating the decomposition of the underlying xylem in some species [17, 18]. In contrast, bark may hinder the colonization of xylem in small branches or twigs (≤2 cm in diameter) by physically excluding insects (potential vectors of microorganisms) and reducing oxygen entry, which may result in a slowdown of the decomposition [10, 11]. These contrasting effects suggest that bark presence might be associated with a trade-off between microclimatic buffering and colonization limitation. Furthermore, in a previous study [17], the authors examined the decomposition of five wood species (5 cm in diameter) and showed that bark accelerated the decomposition rates of three species (*Cunninghamia lanceolata* (Lamb.) Hook., *Toona ciliata* M. Roem., and *Tectona grandis* L. f.), but had no effect on the rate of decomposition in the other two species (*Dipterocarpus turbinatus* C.F. Gaertn. and *Kleinhovia hospita* L.). Similarly, Zhang *et al.* [18] found that bark had a positive effect on the decomposition rate of the xylem of *Betula papyrifera* Marshall (7–9 cm in diameter). Whereas, Jones *et al.* [11] found that bark presence slowed down decomposition in *T. grandis.* This species was one of the five studied in Dossa *et al.* [17], but the branches were smaller (2 cm in diameter). Nevertheless, the mechanisms underlying bark effects on underlying wood decomposition remain poorly understood. One possible pathway of bark effects may be related to the interactions between bark and the microbial communities involved in wood decomposition.

Among the microbial community, fungi are the principal decomposers of lignocellulose capable of degrading lignin, cellulose, and hemicellulose that constitute >90% of wood mass [19–27]. The saprotrophic fungi are a subgroup of fungi that are functionally diverse, encompassing three main decay guilds, white rot, brown rot, and soft rot [23, 28]. All of these functional groups possess the capability to degrade cellulose and hemicellulose, but white rot Basidiomycetes possess ligninolytic peroxidases that fully mineralize lignin [23, 29].

Although fungi dominate lignocellulose decomposition, they rarely act alone. Bacteria often co-occur with fungi, colonizing decayed wood and contributing to nitrogen fixation, lignin demethylation, and secondary metabolite cycling [27, 30]. Bacteria can facilitate fungal activity by altering wood permeability and providing essential nutrients yet may also compete with fungi for labile carbon. Moreover, macro- and mesofauna such as termites, beetle larvae, and mites fragment and shred wood and in doing so may inoculate it with microbial symbionts, accelerating decomposition through detritivore–microbe interactions [6, 14]. These interactions suggest that bark’s physical presence, by excluding or admitting certain invertebrates, could modulate microbial community trajectories and decay rates. Despite growing recognition of these complex interactions, relatively few studies have simultaneously examined how bark affects fungal guild composition and diversity [11, 31], fungal functional traits, and bacterial biomass within the same experimental framework. Although existing studies indicated the potential of fungal and bacterial communities in governing differences in wood decomposition rates between bark-covered and bark-uncovered [11, 18], we lack quantitative information of the contribution of these microorganisms in explaining bark effects. Moreover, it is unclear whether bark effects are driven by microbial biomass or only composition.

The mechanisms underlying fungal community assembly during wood decomposition remain poorly understood. Beta diversity partitioning, which decomposes compositional dissimilarity into turnover (*βsim*) and nestedness (*βsne*) components, provides a framework for inferring whether microbial assembly is driven by deterministic environmental filtering or stochastic colonization [32–36]. Nestedness-dominated beta diversity indicates that poorer communities are subsets of richer ones, reflecting selective species gain or loss, consistent with deterministic habitat filtering, while turnover-dominated beta diversity reflects species replacement driven by deterministic or stochastic colonization [33, 35]. Despite its analytical utility, beta diversity partitioning has rarely been applied to wood-inhabiting fungi in relation to bark, leaving it unclear whether bark acts as a deterministic environmental filter that structures community membership, or whether its removal promotes stochastic assembly dynamics.

**Figure 1:**
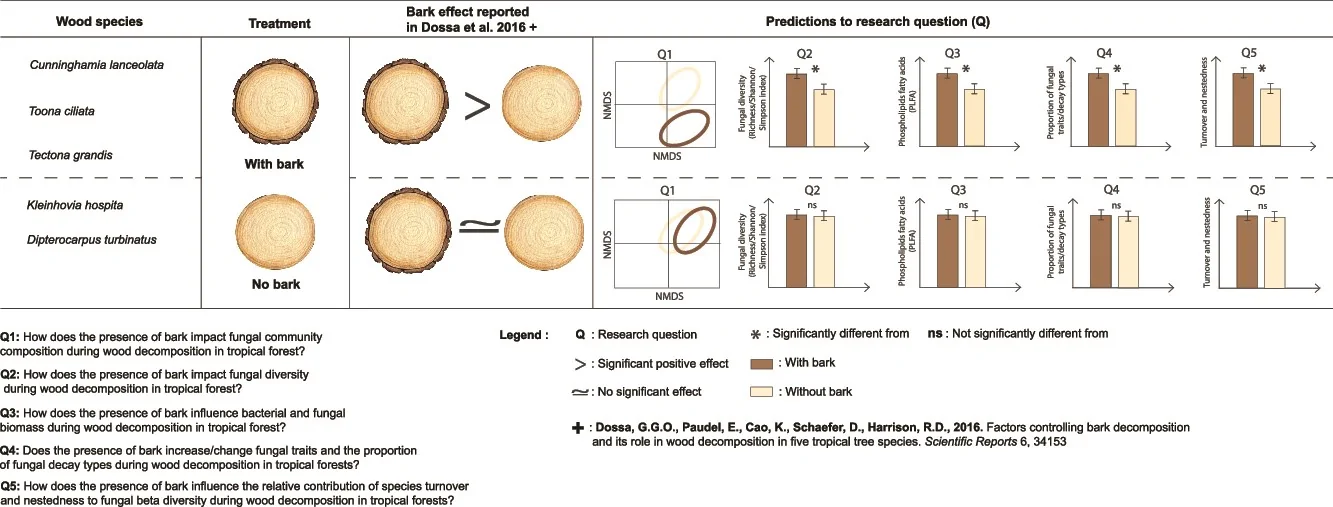
Conceptual framework of this study, illustrating the background of the study, previous results, research questions, and potential predictions.

We address these gaps by integrating high-throughput sequencing and phospholipid fatty acid (PLFA) analyses in a 2-year decomposition experiment involving woody debris with and without bark from five tropical tree species. Building upon earlier work that quantified bark effects on decay rates ([17]; Fig. 1), we investigated whether fungal communities and microbial biomass are associated with those effects. We hypothesized the following: (i) Bark presence alters the fungal community composition during wood decomposition. (ii) Bark presence leads to higher fungal diversity during wood decomposition. (iii) Bark presence leads to increased fungal and bacterial biomass during wood decomposition. (iv) Bark presence increases the expression of certain fungal traits and limits the proportion of fungal decay types during wood decomposition. (v) Bark presence shifts fungal community assembly from turnover-dominated to nestedness-dominated dynamics during wood decomposition. We aim to identify whether bark functions as a biophysical modifier or as a biological filter of microbial decomposers in tropical wood decomposition.

## Materials and methods

### Study site

The experiment was carried out over 2 years from 23 November 2013 to 4 November 2015 in a secondary seasonal rainforest located at 21.9186° N, 101.2680° E (UTM 47Q, 0734280 E, 2425546 N; WGS84), at an elevation of 589 m above sea level (Fig. S1A) [17], which is within the Xishuangbanna Tropical Botanical Garden (XTBG). The region experiences a seasonal climate, with the rainy season occurring between May and October [37, 38]. The dry season is characterized by a foggy and cool-dry period from November to February, followed by a hot-dry period from March to April [37]. The long-term (1959–2017) mean annual temperature is 21.9°C and a mean annual precipitation of 1473 mm [39, 40].

### Bark removal experiment

The experimental design follows Dossa *et al.* [17]. A common garden experiment was employed to reduce environmental variation, with logs (5 cm diameter by 50 cm in length, Fig. S1B) spaced 1 m apart (Fig. S1C). Five tree species (*C. lanceolata*, *D. turbinatus, K. hospita, T. grandis,* and *T. ciliata*) were selected, each subjected to bark removal treatment resulting in wood “With Bark” and “No Bark” (Fig. S1C). Three replicates for each species-treatment combination were implemented. Logs were monitored at five different time intervals: 0, 6, 12, 18, and 24 months, with three logs sampled per species at each interval. At harvest time, the presence or absence of termites was also recorded. Data, including wood specific gravity and mass loss, were measured previously in Dossa *et al.* [17]. Dossa *et al.* [17] found that three species exhibited higher mass loss in the presence of bark, while the remaining species showed no effects of bark. Based on these results, we categorized the five species into two groups, including the bark effect group (*C. lanceolata*, *T. ciliata*, and *T. grandis*) and non-bark effect group (*D. turbinatus* and *K. hospita*) for further analyses in this study (Fig. 1). For DNA analysis, wood dust was collected from the wood up to the pith using a cordless drill (Doug Jones, Doug Jones Jiwei company, Taiwan), preserved in a tube (Corning tube Corning®) and immediately stored in −20°C freezer. Additionally, at each of the three sampling times used for the fungal and biomass datasets (0, 18, and 24 months), disk samples were collected at the base of each harvested tree log immediately upon harvest, prior to drying and grinding for chemical analysis, using an electric hand saw and stored in −20°C freezer. During the wood dust and disk collection, we used ethanol and a flame to sterilize both the drill bit and electric hand saw disc between samples to avoid cross sample contamination (see methods details in supplementary materials).

### Chemical analysis

The chemical properties for both wood “With Bark” and “No Bark” were assessed (Table S1). Disk samples stored in −20°C freezer were dried and ground to a fine powder for the analysis. The following chemical properties were measured: the total carbon (C), nitrogen (N), phosphorus (P), potassium (K), calcium (Ca), iron (Fe), manganese (Mn), copper (Cu), zinc (Zn), water-soluble sugar content, neutral detergent fiber (NDF), acid detergent fiber (ADF), lignin, tannins, and crude fiber were carried out (for details, see supplementary materials).

### Phospholipid fatty acid analysis

Microbial biomass for both wood “With Bark” and “No Bark” was characterized (Table S2) using the modified PLFA method of Frostegård *et al.* [41], as described in Gunina *et al.* [42], Dippold and Kuzyakov *et al.* [43], and Giray *et al.* [44] (for details, see supplementary materials).

### Statistical analysis

Statistical analyses were carried out in R version 4.5.1 (R Development Core Team). To account for sampling biases such as unequal sequencing depth, we followed a two-step procedure. First, as a diagnostic step to assess whether sequencing depth was sufficient to capture fungal diversity, we used a coverage-based rarefaction and extrapolation approach with the package “iNEXT” [45]. Second, to build a single normalized operational taxonomic unit (OTU) table for all downstream community and statistical analyses, we rarefied fungal OTU counts in every sample to a common fixed depth of 4601 reads per sample, the minimum read count observed across all samples.

The bark effect on fungal community composition was tested with permutational multivariate analyses of variance (PERMANOVA) among bark treatments (With Bark versus No Bark) and time (0 months, 18 months, and 24 months) on the Bray–Curtis dissimilarity distances using the function ‘adonis2’ in package “vegan” [46]. Nonmetric multidimensional scaling (NMDS) was used for visualization using the ‘metaMDS’ function of package “vegan” [46]. The differences in community dispersion were tested using dispersion analysis (‘betadisper’) and permutation test on resulting dispersion values [46]. To quantify fungal alpha diversity, we employed Hill numbers where the importance given to species relative abundance increases with Hill order: *q* = 0 for species richness, *q* = 1 for Shannon diversity, and *q* = 2 for Simpson diversity based on the package “iNEXT” [45]. We modeled the alpha diversity across time and bark treatments using Generalized Additive Mixed Models (GAMMs) with the package “gamm4” [47]. To test the bark effect on total, fungal, and bacterial biomass, we also employed GAMM. Beta regression models were fitted using the package “glmmTMB” [48] to determine which fungal guilds were significantly impacted by time and bark treatments. The significance of each fixed effect was evaluated using Type III ANOVA (Wald chi-square tests) with the package “car” [49]. Pairwise contrasts between factor levels were obtained using the package “emmeans” [50] to determine the direction of significant effects. Before fitting the different models, residual normality was checked using the Shapiro–Wilk test combined with visual inspection of residual distributions. For all GAMMs and beta regression models described above, wood species identity was included as a random intercept to account for nonindependence among the multiple logs sampled per species across time and bark treatments.

To understand the ecological processes underlying the assembly of fungi, we partitioned beta diversity into its species turnover (*βsim*) and nestedness (*βsne*) components using the package “betapart” [51]. The partitioning was performed at the OTU level.

Additionally, Spearman correlations among the scaled values of chemical properties were analyzed using the ‘corr.test’ function of package “psych” [52]. The Euclidian distances of the scaled values of dominant fungal taxa, microbial biomass, functional traits, and mass loss percentage were correlated with each of chemical properties using the ‘mantel_test’ function of package “linkET” [53]. *P*-values within each family of tests were further adjusted using the false discovery rate (FDR) correction procedure. The results of the Spearman correlation analyses and Mantel tests were visualized using the ‘qcorrplot’ function of package “linkET” [53].

## Results

### Sequence data analysis

In total, 1 960 250 high-quality Internal Transcribed Spacer 2 ribosomal DNA (ITS2 rDNA) reads were clustered into 7174 non-singleton OTUs at a 97% sequence similarity level (Table S3). As read counts varied from 4601 to 74 573 across all 53 samples (Table S3), samples were rarefied to 4601 reads per sample, resulting in a normalized dataset of 2354 OTUs, containing 243 853 reads in total (Table S3). Among these OTUs, 821 were exclusively recovered from “No Bark” samples, 633 were exclusively from “With Bark” samples, and 693 were shared between “No Bark” and “With Bark” samples (Fig. S2). Across all fungal OTUs obtained, 2154 OTUs (98.03% of total fungal sequences) were identified into 6 phyla and 200 OTUs into unidentified fungi (1.93%). The fungal community was dominated by Ascomycota (1683 OTUs, 56.97% of total fungal sequences) and Basidiomycota (418 OTUs, 40.90%) (Fig. S3). The decay types were mainly dominated by soft rot (91 OTUs, 4.13%), white rot (70 OTUs, 1.31%), and brown rot (9 OTUs, 0.015%), while the primary lifestyle was dominated by saprotrophs (528 OTUs, 42.50%), followed by plant pathogens (214 OTUs, 7.76%) and ectomycorrhizal fungi (25 OTUs, 0.84%) (Fig. S3). In addition, the rarefaction analyses revealed that species richness (OTUs counts) had not reached a plateau in “With Bark” and “No Bark” samples, suggesting that more samples and deeper sequencing would recover extra OTUs. However, Shannon diversity and Simpson diversity had reached a plateau, indicating that diversity estimates were adequately captured (Fig. S4).

### Bark effect on fungal community composition and alpha diversity

**Figure 2:**
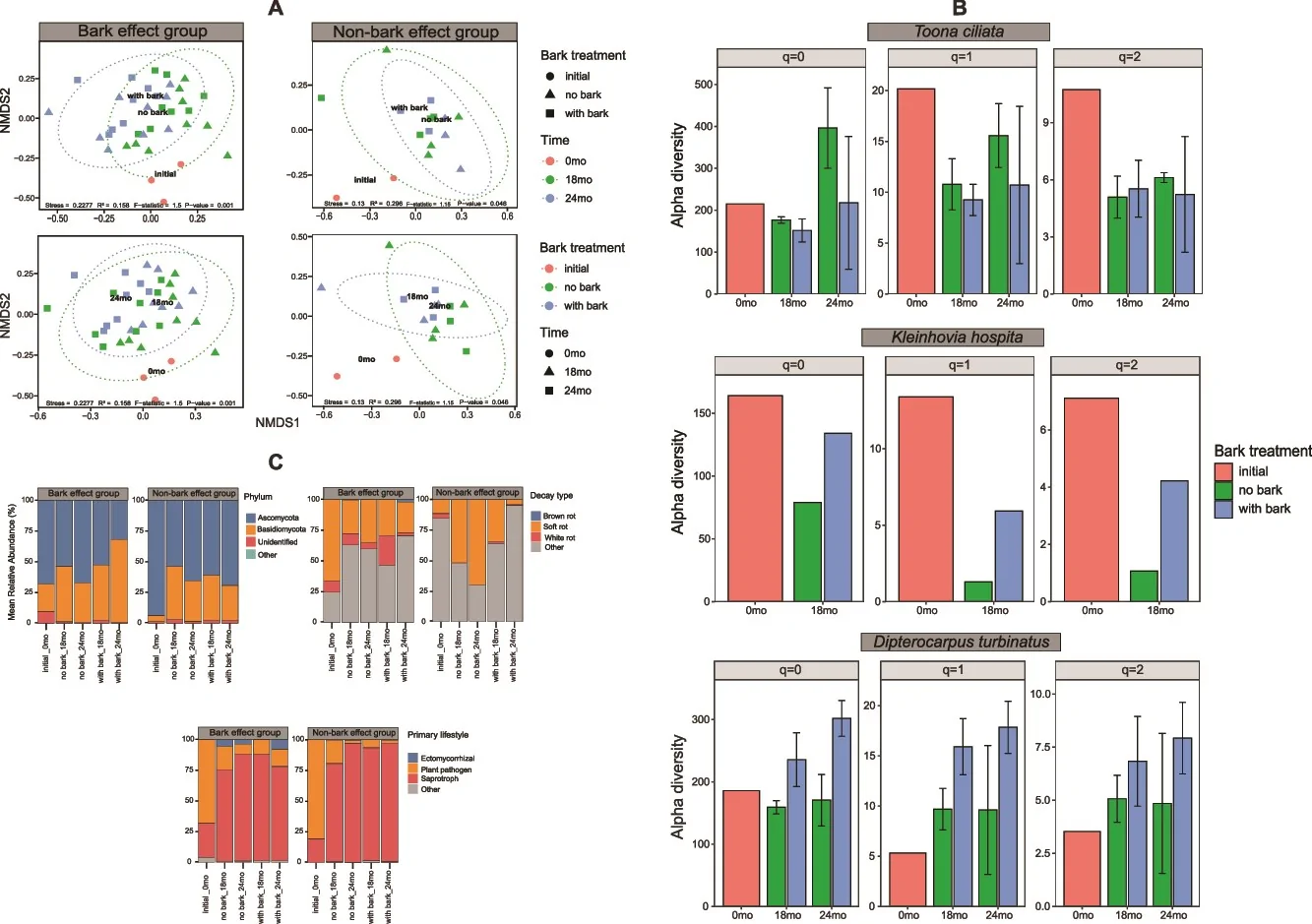
NMDS plots and standard deviation ellipses of fungal community composition for both bark effect and non-bark effect groups (A). Centroids of fungal communities across time and bark treatments are indicated in bold in the ordination. Mean and standard error bars of fungal diversity (richness, Shannon and Simpson's diversity) for *T. ciliata*, *K. hospita*, and *D. turbinatus* (B). Average relative abundance of fungal OTUs assigned to phylum, decay types, and primary lifestyle in bark effect and non-bark effect groups (C).

The fungal community composition showed changes across time with differences between “With Bark” and “No Bark” in both bark effect group and non-bark effect group (Fig. 2A, *P*-values < .05, Table S4). Communities became more homogenous at 18 and 24 months, indicating less variation or a more stable community structure over time. Additionally, fungal communities were less dispersed for “With Bark” compared to “No Bark” (Figs S5 and S6, Table S5) in the bark effect group with statistical nonsignificance in the non-bark effect group. Similar trends recorded from the bark effect group were observed as well in bark effect and non-bark effect groups combined and within all individual species, expect one species, *K. hospita*, due to its fastest decomposition with limited data distinction between “With Bark” and “No Bark” (Figs S5 and S6, Table S5).

Fungal alpha diversity showed no significant changes in richness, Shannon, and Simpson diversity across time and bark treatments for both bark effect group and non-bark effect group (Fig. S7, Table S6). We also found no changes in richness, Shannon, and Simpson diversity across time and bark treatments for bark effect and non-bark effect group combined, *C. lanceolata* and *T. grandis* (Fig. S7, Table S6). However, we observed a decline at 18 months (Fig. 2B, Simpson *P* = .046, Table S6), a marginal significant decline at 24 months (Fig. 2B, Simpson *P* = .082, Table S6), and marginal significant declines in both “With Bark” and “No Bark” in fungal diversity for *T. ciliata* (Fig. 2B, Simpson *P* = .058, Simpson *P* = .064, Table S6). We also found significant declines in “No Bark” and marginal declines in “With Bark” in fungal alpha diversity for *K. hospita* (Fig. 2B, richness *P* < .001, Shannon *P* = .011, Simpson *P* = .068, richness *P* = .091, Shannon *P* = .1, Table S6). In addition, *D. turbinatus* showed marginal significant increase in fungal diversity across bark treatments with a marginal higher fungal diversity for “With Bark” compared to “No Bark” (Fig. 2B, Shannon *P* = .078, Table S6).

### Wood-inhabiting fungi in the bark effect group

The phyla with the highest relative abundance were Ascomycota and Basidiomycota across all the samples. Ascomycota exhibited significant increase for “No Bark” compared to “With Bark” regardless time factor (Fig. 2C, Fig. S8, Table S7). Basidiomycota was in lower proportions in “No Bark” across time compared to “With Bark” and having an increase at 24 months in “With Bark” (Fig. 2C, Fig. S8, Table S7). For decay types, soft rot fungi showed the highest relative abundance across all samples compared to white rot fungi and brown rot fungi (Fig. 2C). However, no changes were observed in soft rot fungi, white rot fungi, and brown rot fungi across time and bark treatments (Fig. 2C, Fig. S8, Table S8). Although no significant changes were detected across time and bark treatments, notable trends were observed for white rot fungi. White rot fungi appeared to be relatively more abundant in “With Bark” compared to “No Bark” at 18 months before decreasing at 24 months. For the primary lifestyle, saprotrophs showed the highest relative abundance but no significant changes across time and bark treatments (Fig. 2C, Fig. S8, Table S9). Plant pathogens were in lower proportions with a reduction across time (from 18 to 24 months) regardless of bark treatments (Fig. 2C, Fig. S8, Table S9). However, there were no changes in ectomycorrhizal fungi across time and bark treatments (Fig. 2C, Fig. S8, Table S9).

### Wood-inhabiting fungi in the non-bark effect group

Ascomycota and Basidiomycota were the phyla with the highest relative abundance, but with no changes across time and bark treatments (Fig. 2C, Fig. S8, Table S7). No changes were also observed in soft rot fungi and brown rot fungi across time and bark treatments (Fig. 2C, Fig. S8, Table S8). However, a change was detected in white rot fungi across bark treatments regardless of time. Although no significant changes were detected across time and bark treatments, notable trends were observed for soft rot fungi. Soft rot fungi appeared to be relatively more abundant in “No Bark” compared to “With Bark” at 18 and 24 months. For the primary lifestyle, saprotrophs showed the highest relative abundance across all the samples regardless of time and bark treatments (Fig. 2C, Fig. S8, Table S9). Plant pathogens in lower proportions followed by ectomycorrhizal fungi exhibiting the lowest proportions without significant changes across time and bark treatments (Fig. 2C, Fig. S8, Table S9).

### Bark effect on fungal and bacterial biomass (PLFA)

**Figure 3:**
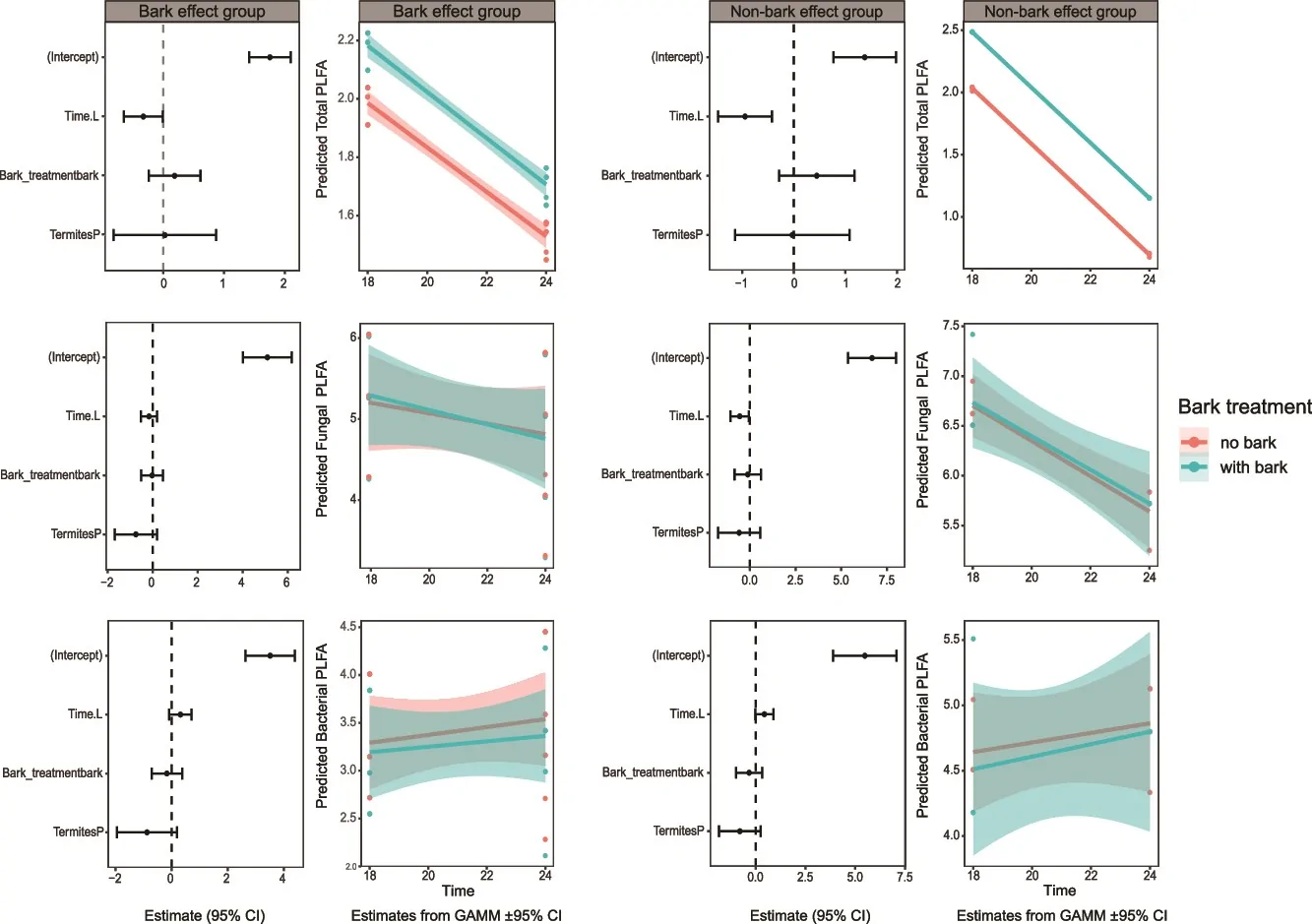
GAMMs for total, fungal, and bacterial biomass under bark treatments and time for bark effect and non-bark effect groups.

There was no effect of bark on total (fungal and bacterial) biomass, fungal biomass, and bacterial biomass for both bark effect and non-bark effect groups (Fig. 3, Tables S10–S16). However, we found a reduction in total (fungal and bacterial) biomass (GAMM, *P* = .044, Table S11) across time for the bark effect group (Fig. 3). Likewise, we found a reduction in total (fungal and bacterial) biomass (GAMM, *P* < .001, Table S14) and fungal biomass (GAMM, *P* = .030, Table S15) as well as a marginal increase in bacterial biomass (GAMM, *P* = .061, Table S16) across time for the non-bark effect group (Fig. 3).

### Temporal dynamics of fungal community turnover and nestedness under bark treatments

**Figure 4:**
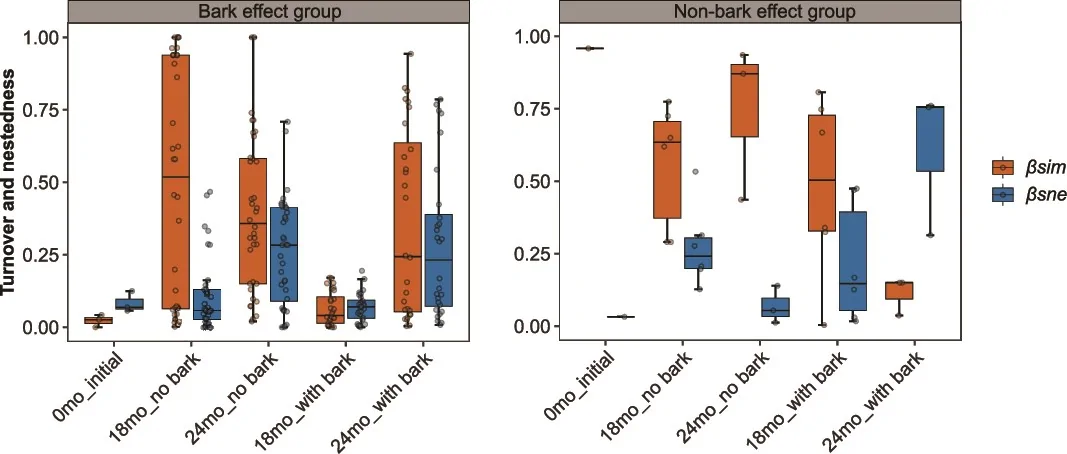
Temporal changes in fungal beta diversity (turnover and nestedness) across bark treatments for bark effect and non-bark effect groups.

In the bark effect group, *βsim* consistently exceeded *βsne* across all conditions and time points, indicating that turnover was the dominant component of beta diversity throughout (Fig. 4, Table S17). Turnover was strongest in “No Bark” at 18 months (*βsim* = 0.489 > *βsne* = 0.110) and 24 months (*βsim* = 0.392 > *βsne* = 0.263) and remained the dominant component even in “With Bark” at 18 months (*βsim* = 0.064 ≈ *βsne* = 0.069) and 24 months (*βsim* = 0.359 > *βsne* = 0.287), although values were substantially lower than in “No Bark” (Fig. 4, Table S17). In the non-bark effect group, *βsim* similarly dominated in “No Bark” at both 18 months (*βsim* = 0.558 > *βsne* = 0.276) and 24 months (*βsim* = 0.747 > *βsne* = 0.069) and in “With Bark” at 18 months (*βsim* = 0.482 > *βsne* = 0.214) (Fig. 4, Table S17). However, the only exception was restricted to “With Bark” at 24 months, where nestedness emerged as the dominant component (*βsne* = 0.610 > *βsim* = 0.112) (Fig. 4, Table S17).

### Wood physicochemical correlates of fungal community composition

**Figure 5:**
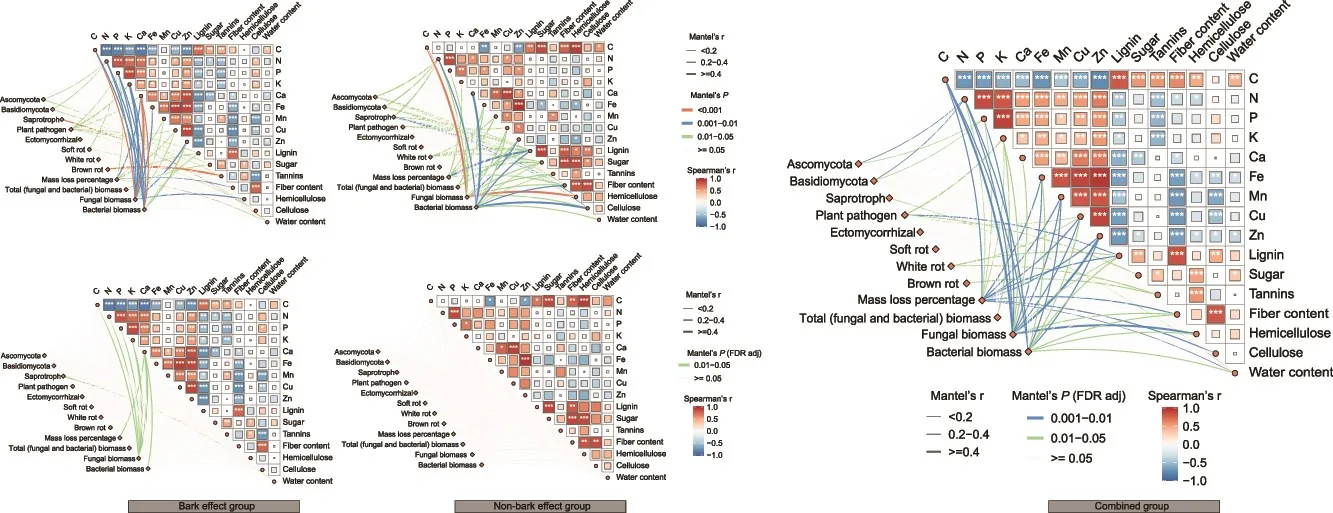
Correlations between fungal community composition and wood physicochemical properties in bark effect, non-bark effect, and combined groups, without and with FDR correction.

The fungal community composition showed significant positive correlations with several wood physicochemical properties, especially properties widely linked to wood decomposition, including carbon, nitrogen, lignin, hemicellulose, cellulose, tannin, and water content in both bark and non-bark effect groups. However, few significant correlations with wood physicochemical properties within either the bark effect group or the non-bark effect group, whereas substantially more significant correlations emerged in combined group after FDR correction (Fig. 5, Table S18). More details are provided in the result section of the supplementary materials.

## Discussion

Our study highlighted the role of bark in shaping fungal communities during decomposition process in a tropical ecosystem for five tree species categorized into bark effect and non-bark effect groups. We also explored whether fungal communities alone are the main factors associated with the bark effects during xylem decomposition.

### Wood-inhabiting fungi differed across time and bark treatments

Ascomycota, including soft rot fungi, showed the highest relative abundance, followed by Basidiomycota, including white rot fungi and brown rot fungi, with some changes in relative abundance across time and bark treatments, aligning with previous decomposition studies [54, 55]. Ascomycota species typically present greater ecological plasticity, allowing them to adapt more effectively to challenging conditions than Basidiomycota species [56–59]. In contrast, Basidiomycota species generally exhibit low abundance in healthy or aging plant tissues. They tend to break down more resistant tissues at early or later stages of microbial succession [60, 61]. Soft rot fungi higher relative abundance in the absence of bark may reflect the contrasting substrate preferences of different fungal decay guilds. They are primarily cellulolytic organisms that preferentially decompose cellulose and hemicellulose rather than lignin [62, 63]. Thus, the presence of bark (enriched with lignin) may create relatively unfavorable conditions for soft rot fungi, potentially reducing their relative abundance. Also, brown rot fungi exhibited the lowest relative abundance compared to white rot fungi and soft rot fungi, perhaps reflecting the lack of competitive abilities of brown rot fungi versus white rot fungi [64–66].

Bark coverage influences the physical properties of dead wood, such as moisture or temperature, creating favorable conditions for certain fungi [14, 15]. Bark traits affected the composition of wood-inhabiting fungi and thereby indirectly affected decomposition rates [16, 67]. Ascomycetes showed higher relative abundance in the absence of bark, while Basidiomycetes, although present in lower proportions, exhibited higher relative abundance in the presence of bark. Although both phyla were positively correlated with nitrogen content, and additionally with water content in the bark effect group and with hemicellulose and cellulose content in the non-bark effect group, the differences in their relative abundance under bark presence may nonetheless be attributed to the moisture retention and nutrient availability maintained by bark, which likely creates conditions favoring Basidiomycota species over Ascomycota species over time. This is further supported by the positive correlation between water content and several chemical properties (carbon, nitrogen, lignin, and cellulose content) and wood mass loss, particularly when bark effect and non-bark effect groups were combined, confirming that moisture and nutrient availability are key regulators of fungal decomposer activity [68, 69]. While shift in fungal communities in relation to bark coverage was supported by Hagge *et al.* [15], Jones *et al.* [11] reported small differences in fungal community composition with no significant substrate (bark, exposed wood, and covered wood) effects subsequently regardless of taxonomic resolution. The observed increase in white rot fungi in the presence of bark, followed by its decline during the decomposition course, corroborates previous studies suggesting that white rot dominates the early stages of decay [23, 70]. This pattern is likely associated with the high lignin content characteristic of both bark and the underlying wood, as our findings revealed a positive correlation between white rot fungi relative abundance and lignin content. Given that white rot fungi are specialized ligninolytic decomposers, their relative abundance correlates with lignin availability. Consequently, as lignin becomes depleted at the early stages of wood decay, this may reduce the relative abundance of white rot fungi leaving space to other decay types to thrive in this evolving environment. Collectively, these findings suggest that lignin availability represents a key determinant of wood decomposition dynamics, fueling white rot fungal activity especially at early decay stages [23, 29], as further confirmed by the positive correlation between lignin content and wood mass loss in bark effect, non-bark effect, and combined groups. Here, saprotrophs showed the highest relative abundance with plant pathogens and ectomycorrhizal fungi remaining consistently low across time regardless bark treatments during wood decomposition, but Dossa *et al.* [7] reported an increase in the relative abundance of these three fungal primary lifestyles with time during the decomposition process of wood species. Our findings contradicted the general expectations that bark presence would promote the relative abundance of saprotrophs, plant pathogens, and ectomycorrhizal fungi. Instead, here the high relative abundance of saprotrophs was associated with various physicochemical properties, including nitrogen, lignin, hemicellulose, cellulose, and water content, suggesting that substrate and moisture availability, rather than the presence of bark, maintain the high relative abundance of saprotrophs throughout the decomposition process [16, 67]. Generally, several of these correlations between fungal taxa/decay types and wood physicochemical properties were not recovered when the bark effect and non-bark effect groups were analyzed separately after FDR correction, likely reflecting a combination of reduced statistical power within each smaller group and confounding by species identity. Overall, our findings partially support our hypothesis (iv) as bark enriched Basidiomycota and white rot fungi in association with lignin content, while having no significant effects on overall decay type proportions or primary lifestyle guilds.

### Bark presence in relation to fungal community composition and alpha diversity

Regardless of bark effects, there was a difference in the community composition of fungi colonizing deadwood of woody plant species in both bark effect and non-bark effect groups. This is in line with our hypothesis (i) suggesting that bark presence altered the fungal community. Similar findings were reported from previous studies of early decomposition (11 months) of three tropical wood species in Panama [11] and for the beech log decomposition located in southeastern Germany [31]. While Hagge *et al.* [15] supported that bark coverage impacted fungal community composition, they found no difference in fungal alpha diversity with bark coverage. Contrary to our hypothesis (ii), we found similar trends, where fungal alpha diversity showed no significant changes over time or across bark treatments in both bark effect and non-bark effect groups. However, certain species taken individually did exhibit notable shifts in fungal alpha diversity across time and bark treatments, partially supporting our hypothesis (ii) at the species level. For instance, *T. ciliata* showed significant reduction in fungal alpha diversity across time and bark treatments. Moreover, *K. hospita* showed significant reduction in fungal alpha diversity across bark treatments with higher fungal diversity in “With Bark” compared to “No Bark,” while *D. turbinatus* showed marginal significant increase across bark treatments with marginal higher fungal diversity in “With Bark” compared to “No Bark.” However, since *T. ciliata*, *K. hospita,* and *D. turbinatus* share some common chemical composition in their tissues, this implies that the overall response of fungal communities may be influenced not only by chemical composition but also by plant species-specific factors. Previous studies have suggested that shift in fungal community composition and diversity could also be linked to host tree specificity [71–75] associated to other factors, including bark coverage and traits [18, 67].

### Fungal and bacterial biomass dynamic across time and bark coverage

Contrary to our hypothesis (iii), bark presence had no significant effect on total (fungal and bacterial) biomass, nor on fungal or bacterial biomass individually, in both bark effect and non-bark effect groups. This indicates that although bark presence shapes fungal community composition and diversity (in some cases), it does not lead to changes in overall fungal or bacterial biomass. Instead, our results showed that fungal and bacterial biomass dynamics were linked to the availability of chemical properties, though the specific correlates and their robustness varied by metric: fungal biomass was most consistently linked to carbon and nitrogen content, whereas bacterial and total (fungal and bacterial) biomass showed broader raw correlations that generally did not survive FDR correction within the individual subgroups, remaining significant mainly in the combined dataset, consistent with the pattern noted above for fungal community composition. Thus, the temporal variations in biomass in both bark effect and non-bark effect groups, with a significant reduction in total (fungal and bacterial) biomass and fungal biomass, alongside an increase in bacterial biomass across time, can be explained by microbial succession involving a shift from initial fungal dominance to a later stage of bacterial dominance. Indeed, fungi seems to be more abundant and effective at breaking down complex organic molecules [22, 27] at the early stages of wood decomposition. Furthermore, since bacterial biomass were strongly correlated with water content, these environmental conditions probably enable bacterial communities to thrive on the resulted simpler compounds. Hu *et al.* [76] also showed similar shift from fungi-dominated at early stages (low wood moisture <20%) to bacteria-dominated at later stages (higher wood moisture) during Chinese fir decomposition.

### Turnover-dominated fungal community assembly amplified by bark removal

In both bark effect and non-bark effect groups, turnover (*βsim*) consistently dominated across nearly all time points and bark treatments, indicating that species replacement was the primary driver of fungal community change throughout decomposition. This finding is consistent with Lepinay *et al.* [77] and Brabcova *et al.* [24], who have demonstrated that wood-inhabiting fungal communities exhibit high levels of spatial variability and beta diversity, with community composition changing substantially as decomposition progresses. Bark removal further amplified this pattern, producing the highest fungal turnover values in both bark effect and non-bark effect groups. Indeed, exposed conditions may intensify microclimatic fluctuations, thereby favoring the selection of certain fungal species to thrive within such conditions. Hagge *et al.* [15] similarly demonstrated that bark coverage determines the assembly processes of wood-inhabiting microorganisms, with deterministic processes most important in completely debarked trees, a pattern consistent for fungi and bacteria. A recent study by Oechler *et al.* [31] further confirmed that bark retention exerted a stronger effect on microbial beta diversity than soil contact or canopy cover, with debarking narrowing phylogenetic diversity through abiotic selection of species-rich but functionally constrained lineages. The emergence of nestedness (*βsne*) dominance at 24 months in “With Bark” in the non-bark effect group indicates a shift toward habitat-filtered, deterministic assembly at the advanced decay stage, whereby bark sustains stable moisture microenvironment, which is consistent with progressive and selective species loss, leaving a structured subset of an earlier, more species-rich community. Overall, our findings partially support our hypothesis (v) as bark presence shifts community assembly from turnover-dominated to nestedness-dominated dynamics only at the advanced stage of wood decomposition (24 months) in the non-bark effect group, while turnover remained dominant across nearly all time points and bark treatments.

## Conclusion

Our study showed that (i) bark plays a crucial role in fungal assemblage during wood decomposition; turnover was the dominant component of beta diversity across nearly all conditions and time points in both bark effect and non-bark effect groups, with higher turnover dynamics for wood without bark; (ii) although the presence of bark affects the composition and diversity of fungal communities, it does not result in changes to the overall fungal and bacterial biomass; and (iii) the shift in fungal communities over time and the variability in species-specific responses suggest that the total effect of bark on wood decomposition may not depend solely on fungal activities but may also be associated with wood physicochemical properties such as lignin, nitrogen, cellulose, and water content, which were significantly associated with both fungal community dynamics and wood mass loss. Future studies should investigate additional biotic factors such as fungal wood endophytes, detritivore abundance, and invertebrate activity, as well as their interactions with chemical and environmental conditions, to more fully understand the bark effects on overall decomposition rates and dynamics in tropical forests.

## Supplementary Material

Clean_revised_version3_Supplemental_Material_Final_ycag258

## Data Availability

Data and code used in this manuscript are available at Zenodo repository (https://zenodo.org/records/21927618). The raw sequence data generated in the scope of this study have been deposited in the NCBI Sequence Read Archive (SRA) under BioProject accession number PRJNA1506057 (BioSamples SAMN62108885–SAMN62108937).
