# Supplementary material for: Bark presence shifts fungal guild composition with varying effects on fungal diversity and biomass in a tropical forest
Source: ISME Commun. 2026 Sep 6;6(1):ycag258. doi: 10.1093/ismeco/ycag258 (PMC13602270; doi:10.1093/ismeco/ycag258)
Supplement: Supplementary file 1 [file clean_revised_version3_supplemental_material_final_ycag258.docx]

***Bark presence shifts fungal guild composition with varying effects on fungal diversity and biomass in a tropical forest***

***Short running title: Bark shapes wood fungal assemblages***

Jean Evans Israel Codjia^1,2,3^ ([evans_codjia@netcourrier.com](mailto:evans_codjia@netcourrier.com)), Busayo Joshua Babalola^4,5^ ([busayobabalola@gmail.com](mailto:busayobabalola@gmail.com)), Weiming Hu^6^ ([huweiming2009@gmail.com](mailto:huweiming2009@gmail.com)), Yun-Qiang Yang^2^ ([yangyunqiang@xtbg.ac.cn](mailto:yangyunqiang@xtbg.ac.cn)), Sewanou H. Honfo^7^ ([honfosewanou@gmail.com](mailto:honfosewanou@gmail.com)), Ekananda Paudel^8^ ([en.paudel@outlook.com](mailto:en.paudel@outlook.com); [en.botany@gmail.com](mailto:en.botany@gmail.com)), Douglas Schaefer^9^ ([schaefer@mail.kib.ac.cn](mailto:schaefer@mail.kib.ac.cn)), Kun-Fang Cao^10^ ([kunfangcao@gxu.edu.cn](mailto:kunfangcao@gxu.edu.cn)), Jian-Chu Xu^9,11^ ([jxu@mail.kib.ac.cn](mailto:jxu@mail.kib.ac.cn)), Yong-Ping Yang^2^ ([yangyp@xtbg.ac.cn](mailto:yangyp@xtbg.ac.cn)), Rhett D. Harrison^12^ ([R.Harrison@cifor-icraf.org](mailto:R.Harrison@cifor-icraf.org)), Gbadamassi G.O. Dossa^1^ * ([dgbadamassi@gmail.com](mailto:dgbadamassi@gmail.com))

^1^Yunnan Key Laboratory of Forest Ecosystem Stability and Global Change, Xishuangbanna Tropical Botanical Garden, Chinese Academy of Sciences, Menglun, Mengla, Yunnan 666303, China

^2^Yunnan International Joint Laboratory for the Conservation and Utilization of Tropical Timber Tree Species, Xishuangbanna Tropical Botanical Garden, Chinese Academy of Sciences, Mengla, Yunnan, 666303, China

^3^Research Unit Tropical Mycology and Plant-Soil Fungi Interactions, Faculty of Agronomy, University of Parakou, BP 123, Parakou, Benin

^4^Department of Plant Biology and Plant Pathology, University of Georgia, Athens, Georgia, 30602, USA

^5^Division of Natural Sciences, Oglethorpe University, Atlanta, Georgia, USA

^6^Division of Gastroenterology, Hepatology and Nutrition, Children’s Hospital of Philadelphia, Philadelphia, PA 19104, USA

^7^Division of Statistics, Montreal Health Innovations Coordinating Center (MHICC), Montreal Heart Institute, Université de Montréal, Montreal, Canada

^8^Nepal Academy of Science and Technology, Khumaltar, Lalitpur, Nepal

^9^Centre for Mountain Futures, Kunming Institute of Botany, Chinese Academy of Science, Kunming 650201, Yunnan, China

^10^Ecophysiology and Evolution Group, Guangxi Key Laboratory of Forest Ecology and Conservation, and College of Forestry, Guangxi University, Nanning, China

^11^Key Laboratory of Economic Plants and Biotechnology, Kunming Institute of Botany, Chinese Academy of Sciences, Kunming, 650201, China

^12^Landscape Alliance, Lusaka, Zambia

* **Corresponding author physical mailing address:**

**Gbadamassi G.O. Dossa**

Yunnan Key Laboratory of Forest Ecosystem Stability and Global Change, Xishuangbanna Tropical Botanical Garden, Chinese Academy of Sciences,

Menglun, Mengla, Yunnan 666303, China.

* **Corresponding author email address:** [dgbadamassi@gmail.com](mailto:dgbadamassi@gmail.com)

**Supplementary information materials**

**Materials and methods**

**DNA extraction, high-throughput sequencing and bioinformatics**

The methodology follows Dossa et al. [1]. Briefly, DNA was extracted from 150 to 250 mg of homogenized wood dust sample using the ZR Soil Microbe DNA MiniPrep kit (Zymo Research, Irvine, CA, USA) according to the manufacturer’s instructions (Fig. S1D). Polymerase chain reaction (PCR) amplification was performed at the internal transcribed spacer region (ITS), using the primer pair ITS1F/ ITS2 [2]. Purified PCR products were then pooled and sequenced using 2 x 300 bp paired-end sequencing on Illumina MiSeq platform. The program MOTHUR [3] was used to pair sequences, generating contigs and excluding those with less than 100 bp overlap. The filtered sequences were then processed in QIIME [4], for chimera removal using USEARCH6.1 pipeline [5]. Operational taxonomic units (OTUs) were identified *de novo* at 97% similarity, with taxonomy assignment based on the UNITE database [6]. FungalTraits database was used to map OTUs to their respective traits (taxonomic diversity, primary lifestyle and decay type) [7]. Read counts were interpreted as semi-quantitative estimates of relative fungal abundance, consistent with prior studies showing that ITS metabarcoding read abundance correlates with independently measured fungal abundance [8, 9]. However, PCR and sequencing biases, including ITS copy-number variation, could affect precision at the taxon level [10, 11]; abundances were therefore interpreted as relative and comparative across treatments rather than absolute.

**Chemical analysis**

Several equipment were used for the different measurements, such as: (1) a Vario Max CN element analyzer (Elementa Analsensysteme, Germany) for C and N contents, (2) a coupled plasma atomic emission spectrometer (iCAP 6300, IRIS Advantage, E R, Thermo Fisher Scientific, USA) for P and K contents, (3) an Analyzer 3 (SEAL Analytical GmbH, Germany) for water-soluble sugar content, (4) a Fibertec ™ 2010 (FOSS Analytical AB, Sweden) for NDF, ADF, ADL, and fiber contents. Additionally, NDF, ADF and lignin were later used to determine hemicellulose and cellulose contents based on the following equations:

$Hemicellulose=NDF-ADF$	(1), where:

- NDF is neutral detergent fiber content (%),

- ADF is acid detergent fiber content (%)

$Cellulose=ADF-Lignin$	(2), where:

- ADF is acid detergent fiber content (%)

- Lignin is lignin content (%) same as acid detergent lignin [ADL (%)]

**Phospholipid fatty acid analysis (PLFA)**

Briefly, the PLFA were extracted from 100 mg of wood dust samples stored at –20 °C using a methanol, chloroform, citric acid solution. The extracts were then purified and separated by solid phase extraction (SPE), saponified and methylated to form fatty acid methyl esters (FAMEs). FAMEs were identified and quantified on an Agilent 6890 gas chromatography (GC, Agilent Technologies, Wilmington, DE, USA) coupled with the MIDI Sherlock Microbial Identification System (MIDI Inc., Newark, DE, USA).

Fatty acids were summed into biomarker groups. Those referring to bacteria include all *iso* and *anteiso* branch chain fatty acids [12], *10-methyl and hydroxy* branched fatty acids as well as *monounsaturated* and *cyclopropane* fatty acids [13–17] while the *18:2ω6,9*, *18:1ω9* and *16:1ω5c* were used as an indicator of fungi [18–21].

**Results**

**Wood-inhabiting fungi relative abundance**

Apart from the bark and non-bark effect groups, we also evaluated the relative abundance of OTUs assigned to phylum, decay types, and primary lifestyle across all the samples for the combined bark effect and non-bark effect groups, as well as for each individual species. Similar trends previously observed in the bark effect and non-bark effect groups regarding the abundance of Ascomycota, Basidiomycota, soft rot fungi, white rot fungi, saprotrophs, plant pathogens, and ectomycorrhizal fungi, were also noted in most cases (Fig. S9).

**Wood physicochemical correlates of fungal community composition in bark effect group**

The fungal community composition showed significant positive correlations with several wood physicochemical properties, especially properties widely linked to wood decomposition, including carbon, nitrogen, lignin, hemicellulose, cellulose, tannin and water content (Fig. 5, Table S18). Thus, at the phyla level, Ascomycota and Basidiomycota were both significantly correlated with nitrogen content (Ascomycota: r = 0.112, p = 0.032; Basidiomycota: r = 0.134, p = 0.013) and water content (Ascomycota: r = 0.102, p = 0.046; Basidiomycota: r = 0.131, p = 0.020). Regarding primary lifestyle, saprotrophs were significantly correlated with water content (r = 0.253, p = 0.001), while plant pathogens showed significant correlations with lignin content (r = 0.150, p = 0.030), tannin content (r = 0.184, p = 0.028), and cellulose content (r = 0.134, p = 0.045). Ectomycorrhizal fungi showed no significant correlations with any primary chemical properties. For decay types, soft rot and brown rot were significantly correlated with cellulose content (r = 0.187, p = 0.034) and tannin content (r = 0.412, p = 0.003), respectively, while white rot showed a marginal correlation with lignin content (r = 0.152, p = 0.051). Furthermore, wood mass loss was significantly correlated with lignin content (r = 0.141, p = 0.026) and water content (r = 0.142, p = 0.049), with marginal correlations with nitrogen content (r = 0.090, p = 0.095) and cellulose content (r = 0.114, p = 0.054). Fungal biomass was strongly correlated with carbon content (r = 0.398, p = 0.001), nitrogen content (r = 0.331, p = 0.002), and hemicellulose content (r = 0.263, p = 0.009). Bacterial biomass was significantly correlated with carbon content (r = 0.259, p = 0.006), nitrogen content (r = 0.223, p = 0.004), and lignin content (r = 0.140, p = 0.049), with a marginal correlation with water content (r = 0.157, p = 0.071). The total (fungal and bacterial) biomass showed no significant correlations with any primary chemical properties.

However, after FDR correction, the fungal community composition showed limited significant correlations (Fig. 5, Table S18). At the phyla level, neither Ascomycota nor Basidiomycota was significantly correlated with any of the primary chemical properties. Regarding primary lifestyle, saprotrophs were significantly correlated with water content (r = 0.253, p = 0.048); plant pathogens and ectomycorrhizal fungi showed no significant correlations with any primary chemical properties. None of the three decay types (soft rot, white rot, brown rot) were significantly correlated with any primary chemical properties, although brown rot showed a marginal association with tannin content (r = 0.412, p = 0.064). Wood mass loss showed no significant correlation with any primary chemical properties. Fungal biomass was significantly correlated with carbon (r = 0.398, p = 0.048) and nitrogen content (r = 0.331, p = 0.048). Bacterial biomass showed no significant correlation with any primary chemical properties. The total (fungal and bacterial) biomass also showed no significant correlations with any primary chemical properties.

**Wood physicochemical correlates of fungal community composition in non-bark effect group**

The patterns of correlation between fungal community composition and wood physicochemical in non-bark effect group differed notably from those observed in bark effect group (Fig. 5, Table S18). Thus, at the phyla level, Ascomycota and Basidiomycota were significantly correlated with nitrogen content (Ascomycota: r = 0.256, p = 0.049; Basidiomycota: r = 0.270, p = 0.034), hemicellulose content (Ascomycota: r = 0.326, p = 0.012; Basidiomycota: r = 0.360, p = 0.013), and cellulose content (Ascomycota: r = 0.357, p = 0.021; Basidiomycota: r = 0.370, p = 0.020). Regarding primary lifestyle, saprotrophs were significantly correlated with nitrogen content (r = 0.251, p = 0.037), lignin content (r = 0.223, p = 0.043), hemicellulose content (r = 0.340, p = 0.006), and cellulose content (r = 0.374, p = 0.004), with a marginal correlation with carbon content (r = 0.206, p = 0.056). Plant pathogens were significantly correlated with lignin content (r = 0.427, p = 0.017), while ectomycorrhizal fungi showed no significant correlations with any primary chemical properties. For decay types, white rot was significantly correlated with lignin content (r = 0.439, p = 0.021), and water content (r = 0.305, p = 0.046), while soft rot and brown rot showed no significant with any primary chemical properties. Furthermore, wood mass loss was significantly correlated with lignin content (r = 0.517, p = 0.009), with a marginal correlation with water content (r = 0.251, p = 0.070). Fungal biomass was strongly correlated with carbon (r = 0.471, p = 0.001), nitrogen content (r = 0.349, p = 0.018), hemicellulose content (r = 0.533, p = 0.001), cellulose content (r = 0.395, p = 0.013), tannin content (r = 0.208, p = 0.044) with a marginal correlation with lignin content (r = 0.214, p = 0.075). Bacterial biomass was significantly correlated with nitrogen content (r = 0.339, p = 0.009), lignin content (r = 0.358, p = 0.008), tannin content (r = 0.196, p = 0.047), cellulose content (r = 0.438, p = 0.003), and water content (r = 0.323, p = 0.018), with a marginal association with hemicellulose content (r = 0.150, p = 0.092). The total (fungal and bacterial) biomass was significantly correlated with lignin content (r = 0.342, p = 0.034), with a marginal correlation with tannin content (r = 0.164, p = 0.092).

However, after FDR correction, no fungal community composition variable (phylum, primary lifestyle, or decay type), wood mass loss, or biomass metric was significantly correlated with any of the primary chemical properties in the non-bark effect group (Fig. 5, Table S18). Among the associations approaching but not reaching significance, fungal biomass showed marginally significant associations with carbon (r = 0.471, p = 0.096) and hemicellulose content (r = 0.533, p = 0.096).

**Wood physicochemical correlates of fungal community composition for combined group**

When all five species were analyzed together (bark effect and non-bark effect groups combined), fungal community composition showed significant correlations with several of the focal wood chemical properties after FDR correction (Fig. 5, Table S18). At the phyla level, both Ascomycota and Basidiomycota were significantly correlated with nitrogen content (Ascomycota: r = 0.177, p = 0.008; Basidiomycota: r = 0.197, p = 0.008). Regarding primary lifestyle, saprotrophs were significantly correlated with water content (r = 0.151, p = 0.032), while plant pathogens were significantly correlated with lignin content (r = 0.278, p = 0.008). Ectomycorrhizal fungi showed no significant correlations with any focal property. For decay types, white rot was significantly correlated with lignin content (r = 0.215, p = 0.035) and tannin content (r = 0.225, p = 0.048), while brown rot was significantly correlated with tannin content (r = 0.383, p = 0.013); soft rot showed no significant correlations with any focal property. Wood mass loss was significantly correlated with carbon, cellulose, lignin, and water content (all p = 0.008), and with nitrogen content (p = 0.013). Fungal biomass was significantly correlated with carbon, nitrogen, and hemicellulose content (all p = 0.008), and with lignin content (p = 0.025). Bacterial biomass was significantly correlated with carbon and nitrogen content (both p = 0.008), hemicellulose content (p = 0.018), lignin content (p = 0.025), and water content (p = 0.035). The total (fungal and bacterial) biomass showed no significant correlation with any focal property. Notably, several of these pooled-species correlations, were not recovered when the bark effect and non-bark effect subgroups were analyzed after FDR correction, likely reflecting a combination of reduced statistical power within each smaller subgroup and confounding by species identity.

**References**

1.	Dossa GGO, Yang Y-Q, Hu W, Paudel E, Schaefer D, Yang Y-P et al. Fungal succession in decomposing woody debris across a tropical forest disturbance gradient. *Soil Biology and Biochemistry* 2021;**155**:108142. https://doi.org/10.1016/j.soilbio.2021.108142

2.	White T, Bruns T, Lee S, Taylor J, Innis M, Gelfand D et al. Ampliﬁcation and direct sequencing of fungal ribosomal RNA genes for phylogenetics. PCR protocols: a guide to methods and applications. Academic Press, California, 1990, 315–322.

3.	Schloss PD, Westcott SL, Ryabin T, Hall JR, Hartmann M, Hollister EB et al. Introducing mothur: open-source, platform-independent, community-supported software for describing and comparing microbial communities. *Applied and Environmental Microbiology* 2009;**75**:7537–7541. https://doi.org/10.1128/AEM.01541-09

4.	Caporaso JG, Kuczynski J, Stombaugh J, Bittinger K, Bushman FD, Costello EK et al. QIIME allows analysis of high-throughput community sequencing data. *Nature Methods* 2010;**7**:335–336. https://doi.org/10.1038/nmeth.f.303

5.	Edgar RC, Haas BJ, Clemente JC, Quince C, Knight R. UCHIME improves sensitivity and speed of chimera detection. *Bioinformatics* 2011;**27**:2194–2200. https://doi.org/10.1093/bioinformatics/btr381

6.	Kõljalg U, Nilsson RH, Abarenkov K, Tedersoo L, Taylor AFS, Bahram M et al. Towards a unified paradigm for sequence-based identification of fungi. *Molecular Ecology* 2013;**22**:5271–5277. https://doi.org/10.1111/mec.12481

7.	Põlme S, Abarenkov K, Henrik Nilsson R, Lindahl BD, Clemmensen KE, Kauserud H et al. FungalTraits: a user-friendly traits database of fungi and fungus-like stramenopiles. *Fungal Diversity* 2020;**105**:1–16. https://doi.org/10.1007/s13225-020-00466-2

8.	Taylor DL, Walters WA, Lennon NJ, Bochicchio J, Krohn A, Caporaso JG et al. Accurate estimation of fungal diversity and abundance through improved lineage-specific primers optimized for illumina amplicon sequencing. *Applied and Environmental Microbiology* 2016;**82**:7217–7226. https://doi.org/10.1128/AEM.02576-16

9.	Wang D, Trimbos KB, Gomes SIF, Jacquemyn H, Merckx VSFT. Metabarcoding read abundances of orchid mycorrhizal fungi are correlated to copy numbers estimated using ddPCR. *New Phytologist* 2024;**242**:1825–1834. https://doi.org/10.1111/nph.19385

10.	Lindahl BD, Nilsson RH, Tedersoo L, Abarenkov K, Carlsen T, Kjøller R et al. Fungal community analysis by high-throughput sequencing of amplified markers – a user’s guide. *New Phytologist* 2013;**199**:288–299. https://doi.org/10.1111/nph.12243

11.	Lofgren LA, Uehling JK, Branco S, Bruns TD, Martin F, Kennedy PG. Genome-based estimates of fungal rDNA copy number variation across phylogenetic scales and ecological lifestyles. *Molecular Ecology* 2019;**28**:721–730. https://doi.org/10.1111/mec.14995

12.	Denef K, Roobroeck D, Manimel Wadu M, Lootens P, Boeckx P. Microbial community composition and rhizodeposit-carbon assimilation in differently managed temperate grassland soils. *Soil Biology and Biochemistry* 2009;**41**:144–153. https://doi.org/10.1016/j.soilbio.2008.10.008

13.	Frostegård Å, Bååth E, Tunlio A. Shifts in the structure of soil microbial communities in limed forests as revealed by phospholipid fatty acid analysis. *Soil Biology and Biochemistry* 1993;**25**:723–730. https://doi.org/10.1016/0038-0717(93)90113-P

14.	Frostegård A, Bååth E. The use of phospholipid fatty acid analysis to estimate bacterial and fungal biomass in soil. *Biology and Fertility of Soils* 1996;**22**:59–65. https://doi.org/10.1007/BF00384433

15.	Zelles L. Phospholipid fatty acid profiles in selected members of soil microbial communities. *Chemosphere* 1997;**35**:275–294. https://doi.org/10.1016/S0045-6535(97)00155-0

16.	Frostegård A, Tunlid A, Bååth E. Use and misuse of PLFA measurements in soils. *Soil Biology and Biochemistry* 2011;**43**:1621–1625. https://doi.org/10.1016/j.soilbio.2010.11.021

17.	Barka EA, Vatsa P, Sanchez L, Gaveau-Vaillant N, Jacquard C, Meier-Kolthoff JP et al. Taxonomy, Physiology, and Natural Products of Actinobacteria. *Microbiology and Molecular Biology Reviews* 2016;**80**:1–43. https://doi.org/10.1128/MMBR.00019-15

18.	Olsson PA, Bååth E, Jakobsen I, Söderström B. The use of phospholipid and neutral lipid fatty acids to estimate biomass of arbuscular mycorrhizal fungi in soil. *Mycological Research* 1995;**99**:623–629. https://doi.org/10.1016/S0953-7562(09)80723-5

19.	Olsson PA. Signature fatty acids provide tools for determination of the distribution and interactions of mycorrhizal fungi in soil. *FEMS Microbiology Ecology* 1999;**29**:303–310. https://doi.org/10.1016/S0168-6496(99)00021-5

20.	Joergensen RG, Wichern F. Quantitative assessment of the fungal contribution to microbial tissue in soil. *Soil Biology and Biochemistry* 2008;**40**:2977–2991. https://doi.org/10.1016/j.soilbio.2008.08.017

21.	Joergensen RG. Phospholipid fatty acids in soil—drawbacks and future prospects. *Biology and Fertility of Soils* 2022;**58**:1–6. https://doi.org/10.1007/s00374-021-01613-w

**Figures**

**Fig. S1.** Study area. (A) Remnant secondary forest within Xishuangbanna Tropical Botanical Garden (XTBG), Menglun, Xishuangbanna, southern Yunnan, China. (B) Wood sampling. (C) Bark removal experiment. (D) Sample preparation for Next-generation sequencing (NGS) and Phospholipid Fatty Acid (PLFA).

**Fig. S2.** Venn diagram displaying the unique and shared fungal species (OTUs) across all samples for “No bark” and “With Bark”.

**Fig. S3.** Percentage of fungal OTUs across all samples.

**Fig. S4.** Rarefaction curves of fungal OTUs (operational taxonomic units) clustered at 97% sequence identity for all samples.

**Fig. S5.** Non-metric multidimensional scaling (NMDS) plots and standard deviation ellipses of fungal community composition for bark effect and non-bark effect groups combined and all individual plant species.

**Fig. S6.** Homogeneity of multivariate dispersions (PERMDISP) of fungal communities across all samples.

**Fig. S7.** Changes in fungal alpha diversity (Richness, Shannon and Simpson diversity) for bark effect and non-bark effect groups, bark effect and non-bark effect groups combined, *Cunninghamia lanceolata* and *Tectona grandis*.

**Fig. S8.** Effect of time and bark treatment on relative abundance of fungal operation taxonomic units (OTUs) in bark effect and non-bark effect groups.

**Fig. S9.** Average relative abundance of fungal operation taxonomic units (OTUs) assigned to Phylum, decay types and primary lifestyle in bark effect and non-bark effect groups combined and all individual plant species.

**Tables**

**Table S1.** Concentrations of chemical properties of the five target plant species across time and treatments

| Sample ID | Species | Time | Bark treatments | Water (%) | C (g/kg) | N (g/kg) | P (g/kg) | K (g/kg) | Ca (%) | Fe (%) | Mn (%) | Cu (%) | Zn (%) | Lignin (%) | Sugar (%) | Tannins (%) | Fiber (%) | Hemicellulose (%) | Cellulose (%) |
| --- | --- | --- | --- | --- | --- | --- | --- | --- | --- | --- | --- | --- | --- | --- | --- | --- | --- | --- | --- |
| X13 | *Kleinhovia hospita* | 0mo | initial | 1.046870748 | 476 | 6.2 | 1.06 | 7.58 | NA | NA | NA | NA | NA | 34.1 | 0.78 | NA | 71.28 | 10.67 | 44.99 |
| X218 | *Cunninghamia lanceolata* | 18mo | with bark | 0.153136557 | 514 | 1.4 | 0.04 | 0.26 | 1.22 | 0.762 | 0.1001 | 0.00145 | 0.0031 | 31.1 | 0.39 | 0.92 | 59.09 | 13.84 | 30.11 |
| X219 | *Cunninghamia lanceolata* | 18mo | with bark | 0.179366954 | 497 | 1.5 | 0.05 | 0.31 | 1.04 | 2.34 | 0.1607 | 0.0025 | 0.002 | 29.7 | 0.32 | 0.68 | 58.09 | 14.67 | 30.19 |
| X224 | *Dipterocarpus turbinatus* | 18mo | with bark | 0.081517353 | 269 | 3.86 | 0.22 | 1.2 | 1.2 | 14.63 | 0.2949 | 0.0066 | 0.0277 | 12.68 | 0.17 | 0.14 | 30.8 | 5.5 | 27.9 |
| X225 | *Dipterocarpus turbinatus* | 18mo | with bark | 0.089216945 | 269 | 3.86 | 0.22 | 1.2 | 1.2 | 14.63 | 0.2949 | 0.0066 | 0.0277 | 12.68 | 0.17 | 0.14 | 30.8 | 5.5 | 27.9 |
| X226 | *Dipterocarpus turbinatus* | 18mo | with bark | 0.097228465 | 333 | 4.69 | 0.3 | 2.78 | 1.45 | 12.3 | 0.3047 | 0.0084 | 0.0232 | 9.88 | 0.25 | 0.17 | 34.02 | 8.11 | 29.05 |
| X23 | *Tectona grandis* | 0mo | initial | 0.713572023 | 487 | 3.37 | 0.23 | 1.56 | NA | NA | NA | NA | NA | 26.39 | 1.17 | NA | 65.66 | 12.17 | 49 |
| X243 | *Kleinhovia hospita* | 18mo | with bark | 0.025474827 | 311 | 6.87 | 0.42 | 1.74 | 7.45 | 11.355 | 0.2243 | 0.0144 | 0.03075 | 0.28 | NA | NA | NA | 2.04 | 5.79 |
| X25 | *Toona ciliata* | 0mo | initial | 0.631053269 | 471 | 2.86 | 0.43 | 2.22 | NA | NA | NA | NA | NA | 20.75 | 0.67 | NA | 67.2 | 16.43 | 54.86 |
| X272 | *Tectona grandis* | 18mo | with bark | 0.150306797 | 474 | 2.9 | 0.12 | 0.85 | 2.16 | 1.839 | 0.0223 | 0.0036 | 0.0047 | 20.72 | 0.12 | 0.21 | 59.64 | 11.55 | 39.35 |
| X273 | *Tectona grandis* | 18mo | with bark | 0.186503183 | 478 | 2.66 | 0.04 | 0.15 | 1.6 | 0.762 | 0.012 | 0.0012 | 0.0008 | 25.51 | 0.15 | 0.23 | 71.51 | 10.07 | 46.4 |
| X274 | *Tectona grandis* | 18mo | with bark | 0.126668415 | 441 | 3.03 | 0.12 | 0.61 | 1.8 | 4.945 | 0.036 | 0.0046 | 0.008 | 20.65 | 0.27 | 0.28 | 61.23 | 10.13 | 39.77 |
| X278 | *Toona ciliata* | 18mo | with bark | 0.100669783 | 445 | 2.71 | 0.21 | 0.67 | 2.67 | 3.303 | 0.0429 | 0.0025 | 0.0054 | 18.39 | 0.27 | 0.37 | 56.93 | 14.6 | 45.05 |
| X279 | *Toona ciliata* | 18mo | with bark | 0.099146362 | 356 | 4.3 | 0.3 | 1.12 | 3.94 | 9.814 | 0.1116 | 0.0086 | 0.0145 | 11.61 | 0.25 | 0.23 | 39.45 | 11.68 | 36.16 |
| X280 | *Toona ciliata* | 18mo | with bark | 0.098125333 | 342 | 4.36 | 0.39 | 3.22 | 3.94 | 11.73 | 0.1858 | 0.0094 | 0.019 | 6.77 | 0.27 | 0.26 | 33.72 | 12.7 | 29.18 |
| X308 | *Cunninghamia lanceolata* | 24mo | with bark | 0.528834356 | 501 | 1.42 | 0.05 | 0.23 | 0.84 | 2.675 | 0.0861 | 0.0035 | 0.0037 | 19.31 | 0.98 | 1.56 | 38.29 | 16.17 | 22.22 |
| X309 | *Cunninghamia lanceolata* | 24mo | with bark | 0.68161435 | 487 | 1.64 | 0.05 | 0.29 | 0.94 | 2.251 | 0.0881 | 0.006 | 0.003 | 20.56 | 0.82 | 0.77 | 33.87 | 14.57 | 24.86 |
| X310 | *Cunninghamia lanceolata* | 24mo | with bark | 0.589567605 | 487 | 1.75 | 0.06 | 0.33 | 0.96 | 6.161 | 0.1042 | 0.0046 | 0.0045 | 22.11 | 0.93 | 1.5 | 46.44 | 14.33 | 25.76 |
| X314 | *Dipterocarpus turbinatus* | 24mo | with bark | 0 | 450 | 3.44 | 0.13 | 0.67 | 1.72 | 4.067 | 0.3852 | 0.0077 | 0.009 | 16.43 | 0.61 | 0.27 | 40.33 | 13.06 | 25.92 |
| X315 | *Dipterocarpus turbinatus* | 24mo | with bark | 0.795345789 | 450 | 3.44 | 0.13 | 0.67 | 1.72 | 4.067 | 0.3852 | 0.0077 | 0.009 | 16.43 | 0.61 | 0.27 | 40.33 | 13.06 | 25.92 |
| X316 | *Dipterocarpus turbinatus* | 24mo | with bark | 1.653039833 | 436 | 4.64 | 0.18 | 0.97 | 2.29 | 5.149 | 0.49033 | 0.0063 | 0.01387 | 13.7 | 0.58 | 0.24 | 36.67 | 11.49 | 26.84 |
| X362 | *Tectona grandis* | 24mo | with bark | 0.387284968 | 447 | 2.87 | 0.21 | 1.39 | 4.86 | 5.227 | 0.1059 | 0.0098 | 0.0143 | 16.29 | 0.51 | 0.29 | 52.35 | 12.81 | 34.19 |
| X363 | *Tectona grandis* | 24mo | with bark | 0.434991205 | 479 | 3.22 | 0.11 | 0.44 | 1.95 | 1.195 | 0.0438 | 0.0029 | 0.003 | 19.79 | 0.19 | 0.27 | 52.74 | 12.96 | 33.26 |
| X364 | *Tectona grandis* | 24mo | with bark | 0.466849607 | 482 | 1.55 | 0.12 | 0.13 | 1.65 | 1.321 | 0.0111 | 0.0022 | 0.0018 | 18.97 | 0.18 | 0.24 | 61.29 | 13.27 | 35.42 |
| X368 | *Toona ciliata* | 24mo | with bark | 1.099168943 | 413 | 5.94 | 0.37 | 1.84 | 5.18 | 5.222 | 0.1923 | 0.00755 | 0.0111 | 11.12 | 0.2 | 0.25 | 38.12 | 13.76 | 32.56 |
| X370 | *Toona ciliata* | 24mo | with bark | 0 | 413 | 5.94 | 0.37 | 1.84 | 5.18 | 5.222 | 0.1923 | 0.00755 | 0.0111 | 11.12 | 0.2 | 0.25 | 38.12 | 13.76 | 32.56 |
| X5 | *Cunninghamia lanceolata* | 0mo | initial | 0.576576577 | 511 | 1.77 | 0.15 | 0.93 | NA | NA | NA | NA | NA | 34.19 | 0.39 | NA | 69.32 | 10.73 | 40.45 |
| X7 | *Dipterocarpus turbinatus* | 0mo | initial | 0.823129252 | 481 | 3.34 | 0.12 | 1.63 | NA | NA | NA | NA | NA | 32.19 | 0.67 | NA | 69.56 | 15.38 | 43.29 |
| XW281 | *Cunninghamia lanceolata* | 18mo | no bark | 0.183118584 | 502 | 1.3 | 0.03 | 0.37 | 0.86 | 1.076 | 0.0872 | 0.0014 | 0.00225 | 22.14 | 0.57 | 1.61 | 49.23 | 14.92 | 26.94 |
| XW282 | *Cunninghamia lanceolata* | 18mo | no bark | 0.17495158 | 500 | 1.76 | 0.05 | 0.25 | 0.86 | 0.845 | 0.1151 | 0.0018 | 0.0004 | 25.67 | 0.52 | 0.93 | 49.21 | 12.75 | 31.23 |
| XW283 | *Cunninghamia lanceolata* | 18mo | no bark | 0.168977086 | 499 | 1.56 | 0.03 | 0.25 | 0.87 | 2.105 | 0.0917 | 0.0017 | 0.0013 | 23.59 | 0.6 | 1.47 | 54.57 | 14.19 | 27.83 |
| XW284 | *Dipterocarpus turbinatus* | 18mo | no bark | 0.05 | 349 | 3.54 | 0.23 | 1.06 | 1.42 | 10.44 | 0.1964 | 0.0073 | 0.0186 | 12.28 | 0.31 | 0.2 | 33.41 | 8.92 | 27.13 |
| XW285 | *Dipterocarpus turbinatus* | 18mo | no bark | 0.045606229 | 363 | 4.49 | 0.22 | 1.57 | 1.26 | 10.16 | 0.1948 | 0.0072 | 0.0174 | 14.57 | 0.29 | 0.18 | 42.06 | 8.22 | 32.05 |
| XW286 | *Dipterocarpus turbinatus* | 18mo | no bark | 0.072727273 | 363 | 4.49 | 0.22 | 1.57 | 1.26 | 10.16 | 0.1948 | 0.0072 | 0.0174 | 14.57 | 0.29 | 0.18 | 42.06 | 8.22 | 32.05 |
| XW289 | *Kleinhovia hospita* | 18mo | no bark | 0.037735849 | NA | NA | NA | NA | NA | NA | NA | NA | NA | NA | NA | NA | NA | NA | NA |
| XW290 | *Tectona grandis* | 18mo | no bark | 0.124929156 | 450 | 3.57 | 0.17 | 1.21 | 2.02 | 3.423 | 0.06745 | 0.00495 | 0.00845 | 20.5 | 0.25 | 0.24 | 54.49 | 11.61 | 36.79 |
| XW291 | *Tectona grandis* | 18mo | no bark | 0.183854145 | 467 | 2.26 | 0.05 | 0.34 | 0.93 | 1.432 | 0.0166 | 0.0015 | 0.0014 | 26.29 | 0.22 | 0.18 | 60.85 | 9.3 | 45.71 |
| XW292 | *Tectona grandis* | 18mo | no bark | 0.16717441 | 473 | 2.82 | 0.11 | 0.4 | 0.93 | 1.299 | 0.0174 | 0.002 | 0.0022 | 26.54 | 0.21 | 0.23 | 67.23 | 10.14 | 45.75 |
| XW293 | *Toona ciliata* | 18mo | no bark | 0.133143008 | 405 | 4.23 | 0.26 | 2.61 | 4.48 | 6.322 | 0.1476 | 0.0078 | 0.0142 | 12.62 | 0.28 | 0.29 | 40.93 | 17.66 | 31.71 |
| XW294 | *Toona ciliata* | 18mo | no bark | 0.165418582 | 439 | 2.54 | 0.15 | 0.79 | 2.62 | 3.01 | 0.0505 | 0.0029 | 0.0059 | 10.59 | 0.29 | 0.25 | 53.97 | 19.62 | 42.01 |
| XW295 | *Toona ciliata* | 18mo | no bark | 0.140461766 | 460 | 3.58 | 0.16 | 0.97 | 2.52 | 1.009 | 0.0323 | 0.0019 | 0.0045 | 12.83 | 0.29 | 0.39 | 53.95 | 21.81 | 39.92 |
| XW371 | *Cunninghamia lanceolata* | 24mo | no bark | 0.246835353 | 508 | 1.37 | 0.02 | 0.27 | 0.61 | 1.856 | 0.053 | 0.00295 | 0.00195 | 15.81 | 0.63 | 1.83 | 39.7 | 15.42 | 19.42 |
| XW372 | *Cunninghamia lanceolata* | 24mo | no bark | 0.25198148 | 498 | 2.08 | 0.06 | 0.29 | 1 | 2.585 | 0.0776 | 0.0059 | 0.0058 | 14.75 | 0.52 | 1.36 | 37.55 | 12.5 | 16.71 |
| XW373 | *Cunninghamia lanceolata* | 24mo | no bark | 0.23971006 | 495 | 1.46 | 0.04 | 0.35 | 0.82 | 3.547 | 0.1082 | 0.0083 | 0.0034 | 18.71 | 0.53 | 1.55 | 42.58 | 10.71 | 20.06 |
| XW374 | *Dipterocarpus turbinatus* | 24mo | no bark | 0.435129367 | 391 | 3.09 | 0.19 | 1.68 | 1.12 | 7.87 | 0.2088 | 0.006 | 0.0119 | 7.35 | 0.2 | 0.23 | 32.47 | 9.83 | 24.69 |
| XW375 | *Dipterocarpus turbinatus* | 24mo | no bark | 2.070614571 | 387 | 6.91 | 0.29 | 0.9 | 2.61 | 9.191 | 0.2465 | 0.0102 | 0.0134 | 3.47 | 0.17 | 0.09 | 16.77 | 6.19 | 14.73 |
| XW376 | *Dipterocarpus turbinatus* | 24mo | no bark | 0.764767039 | 416 | 3.05 | 0.12 | 0.76 | 1.01 | 5.304 | 0.055 | 0.0045 | 0.0078 | 9.67 | 0.26 | 0.19 | 48.34 | 14.33 | 34.91 |
| XW380 | *Tectona grandis* | 24mo | no bark | 1.095051422 | 463 | 3.23 | 0.1 | 0.54 | 2.31 | 3.338 | 0.1309 | 0.0084 | 0.0064 | 13.53 | 0.22 | 0.27 | 41.17 | 10.9 | 24.65 |
| XW381 | *Tectona grandis* | 24mo | no bark | 0.28257947 | 448 | 2.92 | 0.19 | 0.59 | 2.45 | 3.433 | 0.0574 | 0.0063 | 0.0077 | 8.45 | 0.23 | 0.27 | 40.17 | 13.47 | 29.84 |
| XW382 | *Tectona grandis* | 24mo | no bark | 0.367746784 | 481 | 2.16 | 0.12 | 0.66 | 1.39 | 0.758 | 0.0536 | 0.0025 | 0.0025 | 17.56 | 0.03 | 0.36 | 47.2 | 13.73 | 32.37 |
| XW383 | *Toona ciliata* | 24mo | no bark | 0.417037448 | 427 | 2.99 | 0.2 | 0.84 | 3.8 | 4.506 | 0.09395 | 0.0076 | 0.0081 | 4.84 | 0.37 | 0.16 | 27.15 | 14.85 | 24.65 |
| XW384 | *Toona ciliata* | 24mo | no bark | 0.33934231 | 461 | 3.3 | 0.14 | 0.3 | 1.92 | 1.421 | 0.0479 | 0.0029 | 0.0028 | 14.18 | 0.4 | 0.32 | 42.2 | 14.47 | 33.33 |
| XW385 | *Toona ciliata* | 24mo | no bark | 0 | 461 | 3.3 | 0.14 | 0.3 | 1.92 | 1.421 | 0.0479 | 0.0029 | 0.0028 | 14.18 | 0.4 | 0.32 | 42.2 | 14.47 | 33.33 |

**Table S2.** Concentrations of phospholipid fatty acid (PLFA)-based fungal and bacterial biomass of the five target plant species across time and treatments

| Sample ID | Time | Bark treatments | Ratio of Fungi PLFA/Bacteria PLFA | Total Fungi PLFA | Total Bacteria PLFA |
| --- | --- | --- | --- | --- | --- |
| X13 | 0mo | initial | 16.219 | 103.318 | 6.37 |
| X218 | 18mo | with bark | 4.905 | 114.589 | 23.363 |
| X219 | 18mo | with bark | 5.897 | 115.286 | 19.549 |
| X224 | 18mo | with bark | 8.511 | 443.075 | 52.06 |
| X225 | 18mo | with bark | 11.902 | 1218.693 | 102.391 |
| X226 | 18mo | with bark | 27.424 | 829.596 | 30.251 |
| X23 | 0mo | initial | 46.072 | 67.469 | 1.464 |
| X243 | 18mo | with bark | 6.385 | 1401.821 | 219.54 |
| X25 | 0mo | initial | 6.641 | 375.742 | 56.581 |
| X272 | 18mo | with bark | 8.673 | 152.127 | 17.539 |
| X273 | 18mo | with bark | 3.774 | 67.09 | 17.775 |
| X274 | 18mo | with bark | 10.236 | 214.875 | 20.993 |
| X278 | 18mo | with bark | 12.907 | 286.175 | 22.172 |
| X279 | 18mo | with bark | 11.633 | 192.146 | 16.517 |
| X280 | 18mo | with bark | 14.803 | 513.277 | 34.674 |
| X308 | 24mo | with bark | 10.325 | 17.92 | 1.736 |
| X309 | 24mo | with bark | 3.807 | 45.885 | 12.053 |
| X310 | 24mo | with bark | 1.924518123 | 29.04688926 | 15.0930713 |
| X314 | 24mo | with bark | 3.136 | 506.185 | 161.393 |
| X315 | 24mo | with bark | 2.776 | 106.388 | 38.318 |
| X316 | 24mo | with bark | 0.665 | 132.014 | 198.603 |
| X362 | 24mo | with bark | 4.846 | 254.447 | 52.503 |
| X363 | 24mo | with bark | 12.256 | 112.968 | 9.218 |
| X364 | 24mo | with bark | 1.983 | 33.828 | 17.057 |
| X368 | 24mo | with bark | 2.984386658 | 122.7648634 | 41.13570977 |
| X370 | 24mo | with bark | 4.940957192 | 955.2809354 | 193.3392455 |
| X5 | 0mo | initial | 0 | 21.614 | 0 |
| X7 | 0mo | initial | 55.132 | 257.634 | 4.673 |
| XW281 | 18mo | no bark | 0 | 18.506 | 0 |
| XW282 | 18mo | no bark | 2.534 | 94.647 | 37.346 |
| XW283 | 18mo | no bark | 11.405 | 71.644 | 6.282 |
| XW284 | 18mo | no bark | 4.345 | 397.318 | 91.452 |
| XW285 | 18mo | no bark | 9.494 | 518.456 | 54.608 |
| XW286 | 18mo | no bark | 4.652 | 578.157 | 124.292 |
| XW289 | 18mo | no bark | 7.508 | 1517.492 | 202.118 |
| XW290 | 18mo | no bark | 4.729 | 223.647 | 47.292 |
| XW291 | 18mo | no bark | 7.712 | 104.961 | 13.61 |
| XW292 | 18mo | no bark | 5.503 | 104.388 | 18.968 |
| XW293 | 18mo | no bark | 11.595 | 589.935 | 50.88 |
| XW294 | 18mo | no bark | 9.531 | 493.533 | 51.781 |
| XW295 | 18mo | no bark | 12.123 | 762.999 | 62.939 |
| XW371 | 24mo | no bark | 3.127161549 | 14.70099924 | 4.701068048 |
| XW372 | 24mo | no bark | 6.437196673 | 88.72366918 | 13.78296698 |
| XW373 | 24mo | no bark | 1.536894748 | 18.96936902 | 12.34265979 |
| XW374 | 24mo | no bark | 1.952573312 | 102.8082197 | 52.65268097 |
| XW375 | 24mo | no bark | 2.733055984 | 674.2749007 | 246.7109728 |
| XW376 | 24mo | no bark | 3.823220602 | 358.7416861 | 93.8323271 |
| XW380 | 24mo | no bark | 9.49228722 | 502.3461567 | 52.92150828 |
| XW381 | 24mo | no bark | 2.699651416 | 70.52758964 | 26.12470233 |
| XW382 | 24mo | no bark | 9.303143629 | 102.1605713 | 10.98129572 |
| XW383 | 24mo | no bark | 3.270422413 | 270.1324759 | 82.5986499 |
| XW384 | 24mo | no bark | 6.075477423 | 87.79276417 | 14.45034819 |
| XW385 | 24mo | no bark | 0.529463701 | 133.3879898 | 251.9303771 |

**Table S3.** Average number of fungal (operational taxonomic unit, OTUs) sequences across all samples

| Sample ID | Time | Bark treatments | Number of sequences per sample before rarefaction | Number of sequences per sample after rarefaction |
| --- | --- | --- | --- | --- |
| X13 | 0mo | initial | 30569 | 4601 |
| X218 | 18mo | with bark | 34617 | 4601 |
| X219 | 18mo | with bark | 25637 | 4601 |
| X224 | 18mo | with bark | 24073 | 4601 |
| X225 | 18mo | with bark | 44029 | 4601 |
| X226 | 18mo | with bark | 42283 | 4601 |
| X23 | 0mo | initial | 31953 | 4601 |
| X243 | 18mo | with bark | 53318 | 4601 |
| X25 | 0mo | initial | 33339 | 4601 |
| X272 | 18mo | with bark | 56157 | 4601 |
| X273 | 18mo | with bark | 48520 | 4601 |
| X274 | 18mo | with bark | 74573 | 4601 |
| X278 | 18mo | with bark | 52644 | 4601 |
| X279 | 18mo | with bark | 44111 | 4601 |
| X280 | 18mo | with bark | 49276 | 4601 |
| X308 | 24mo | with bark | 18466 | 4601 |
| X309 | 24mo | with bark | 14405 | 4601 |
| X310 | 24mo | with bark | 37428 | 4601 |
| X314 | 24mo | with bark | 35212 | 4601 |
| X315 | 24mo | with bark | 38641 | 4601 |
| X316 | 24mo | with bark | 36875 | 4601 |
| X362 | 24mo | with bark | 21280 | 4601 |
| X363 | 24mo | with bark | 17439 | 4601 |
| X364 | 24mo | with bark | 17316 | 4601 |
| X368 | 24mo | with bark | 13519 | 4601 |
| X370 | 24mo | with bark | 35688 | 4601 |
| X5 | 0mo | initial | 45930 | 4601 |
| X7 | 0mo | initial | 70467 | 4601 |
| XW281 | 18mo | no bark | 30416 | 4601 |
| XW282 | 18mo | no bark | 23724 | 4601 |
| XW283 | 18mo | no bark | 28193 | 4601 |
| XW284 | 18mo | no bark | 26548 | 4601 |
| XW285 | 18mo | no bark | 38507 | 4601 |
| XW286 | 18mo | no bark | 61674 | 4601 |
| XW289 | 18mo | no bark | 27189 | 4601 |
| XW290 | 18mo | no bark | 4601 | 4601 |
| XW291 | 18mo | no bark | 48012 | 4601 |
| XW292 | 18mo | no bark | 53488 | 4601 |
| XW293 | 18mo | no bark | 34110 | 4601 |
| XW294 | 18mo | no bark | 53830 | 4601 |
| XW295 | 18mo | no bark | 46026 | 4601 |
| XW371 | 24mo | no bark | 31493 | 4601 |
| XW372 | 24mo | no bark | 55749 | 4601 |
| XW373 | 24mo | no bark | 32218 | 4601 |
| XW374 | 24mo | no bark | 33282 | 4601 |
| XW375 | 24mo | no bark | 27641 | 4601 |
| XW376 | 24mo | no bark | 30034 | 4601 |
| XW380 | 24mo | no bark | 28118 | 4601 |
| XW381 | 24mo | no bark | 28376 | 4601 |
| XW382 | 24mo | no bark | 26954 | 4601 |
| XW383 | 24mo | no bark | 48700 | 4601 |
| XW384 | 24mo | no bark | 53153 | 4601 |
| XW385 | 24mo | no bark | 40449 | 4601 |
|  |  | **TOTAL** | **1960250** | **243853** |

**Table S4.** PERMANOVA analysis for fungal community composition across all samples

|  | Combined group | | | | | Bark effect group | | | | |
| --- | --- | --- | --- | --- | --- | --- | --- | --- | --- | --- |
|  | DF | Sum of Squares | R² | F-value | P-value | DF | Sum of Squares | R² | F-value | P-value |
| Model | 4 | 2.6436 | 0.12223 | 1.671 | **0.001***** | 4 | 2.3487 | 0.15828 | 1.5043 | **0.001***** |
|  | Non-bark effect group | | | | | *Cunninghamia lanceolata* | | | | |
|  | DF | Sum of Squares | R² | F-value | P-value | DF | Sum of Squares | R² | F-value | P-value |
| Model | 4 | 1.7723 | 0.29569 | 1.1546 | **0.046 *** | 4 | 1.6251 | 0.43396 | 1.3416 | **0.008 **** |
|  | *Toona ciliata* | | | | | *Tectona grandis* | | | | |
|  | DF | Sum of Squares | R² | F-value | P-value | DF | Sum of Squares | R² | F-value | P-value |
| Model | 4 | 1.8153 | 0.42452 | 1.291 | **0.009 *** | 4 | 1.9717 | 0.38859 | 1.2711 | **0.004 **** |
|  | *Dipterocarpus turbinatus* | | | | | *Kleinhovia hospita* | | | | |
|  | DF | Sum of Squares | R² | F-value | P-value | DF | Sum of Squares | R² | F-value | P-value |
| Model | 4 | 1.7699 | 0.39911 | 1.3284 | **0.002 **** | 2 | 0.90328 | 1 | − | − |

**Signif. codes :** 0 ‘***’ 0.001 ‘**’ 0.01 ‘*’ 0.05 ‘.’ 0.1 ‘ ’ 1

**Table S5.** Homogeneity of multivariate dispersions (PERMDISP) of fungal communities across all samples

|  | Combined group | | | | | Bark effect group | | | | |
| --- | --- | --- | --- | --- | --- | --- | --- | --- | --- | --- |
|  | DF | Sum of Squares | Mean of Squares | F-value | P-value | DF | Sum of Squares | Mean of Squares | F-value | P-value |
| Time | 2 | 0.042577 | 0.0212887 | 14.872 | **0.001***** | 2 | 0.083682 | 0.041841 | 25.246 | **0.001***** |
| Bark treatments | 2 | 0.047109 | 0.0235544 | 16.365 | **0.001***** | 2 | 0.086252 | 0.043126 | 25.425 | **0.001***** |
|  | Non-bark effect group | | | | | *Cunninghamia lanceolata* | | | | |
|  | DF | Sum of Squares | Mean of Squares | F-value | P-value | DF | Sum of Squares | Mean of Squares | F-value | P-value |
| Time | 2 | 0.016733 | 0.0083667 | 1.7144 | 0.217 | 2 | 0.230137 | 0.115069 | 21.175 | **0.001***** |
| Bark treatments | 2 | 0.014606 | 0.0073031 | 1.3014 | 0.292 | 2 | 0.238602 | 0.119301 | 30.234 | **0.001***** |
|  | *Toona ciliata* | | | | | *Tectona grandis* | | | | |
|  | DF | Sum of Squares | Mean of Squares | F-value | P-value | DF | Sum of Squares | Mean of Squares | F-value | P-value |
| Time | 2 | 0.280303 | 0.140152 | 52.263 | **0.001***** | 2 | 0.304825 | 0.152413 | 44.602 | **0.001***** |
| Bark treatments | 2 | 0.276144 | 0.13807 | 23.401 | **0.001***** | 2 | 0.314210 | 0.157105 | 83.176 | **0.001***** |
|  | *Dipterocarpus turbinatus* | | | | | *Kleinhovia hospita* | | | | |
|  | DF | Sum of Squares | Mean of Squares | F-value | P-value | DF | Sum of Squares | Mean of Squares | F-value | P-value |
| Time | 2 | 0.273620 | 0.136810 | 66.32 | **0.001***** | 1 | 0.14543 | 0.14543 | − | − |
| Bark treatments | 2 | 0.261130 | 0.130565 | 60.244 | **0.001***** | 2 | 0 | 0 | − | − |

**Signif. codes :** 0 ‘***’ 0.001 ‘**’ 0.01 ‘*’ 0.05 ‘.’ 0.1 ‘ ’ 1

**Table S6.** Variations in fungal alpha diversity (Richness, Shannon, Simpson) based on Generalized Additive Mixed Models (GAMM)

| Combined group | type | Variable | Estimate | Std. Error | z-value | P-value |
| --- | --- | --- | --- | --- | --- | --- |
|  | q=0 | (Intercept) | 5.341374789 | 0.212662284 | 25.11669999 | 3.27E-139 |
|  | q=0 | Time18mo | 0.035936312 | 0.232944168 | 0.154270064 | 0.8773968 |
|  | q=0 | Time24mo | 0.046103207 | 0.234620891 | 0.196500862 | 0.844218158 |
|  | q=1 | (Intercept) | 2.638888234 | 0.296626442 | 8.896335126 | 5.77E-19 |
|  | q=1 | Time18mo | 0.012553172 | 0.324882551 | 0.038639108 | 0.969178122 |
|  | q=1 | Time24mo | -0.195143469 | 0.328304585 | -0.594397637 | 0.552246188 |
|  | q=2 | (Intercept) | 1.936054825 | 0.256182305 | 7.557332375 | 4.11E-14 |
|  | q=2 | Time18mo | -0.098261994 | 0.281692783 | -0.348826807 | 0.727219337 |
|  | q=2 | Time24mo | -0.295532564 | 0.286449293 | -1.031709875 | 0.30220805 |
|  | q=0 | (Intercept) | 5.341374646 | 0.212254 | 25.16501294 | 9.68E-140 |
|  | q=0 | Bark_treatmentno bark | 0.010099806 | 0.232507289 | 0.043438666 | 0.965351856 |
|  | q=0 | Bark_treatmentwith bark | 0.073176247 | 0.234158862 | 0.312506844 | 0.754655363 |
|  | q=1 | (Intercept) | 2.638888401 | 0.298189367 | 8.849706575 | 8.77E-19 |
|  | q=1 | Bark_treatmentno bark | -0.011612645 | 0.326700597 | -0.035545221 | 0.971644988 |
|  | q=1 | Bark_treatmentwith bark | -0.163700367 | 0.329846531 | -0.496292521 | 0.619688042 |
|  | q=2 | (Intercept) | 1.936054817 | 0.25859815 | 7.48673112 | 7.06E-14 |
|  | q=2 | Bark_treatmentno bark | -0.136869834 | 0.284769719 | -0.480633384 | 0.630777084 |
|  | q=2 | Bark_treatmentwith bark | -0.246606729 | 0.288382818 | -0.855136689 | 0.39247548 |
| Bark effect group | q=0 | (Intercept) | 5.443857734 | 0.292161376 | 18.6330507 | 1.73E-77 |
|  | q=0 | Time18mo | 0.020224539 | 0.316883402 | 0.063823282 | 0.94911094 |
|  | q=0 | Time24mo | -0.085333238 | 0.316927173 | -0.269251883 | 0.787735857 |
|  | q=1 | (Intercept) | 2.838755563 | 0.367797928 | 7.718247833 | 1.18E-14 |
|  | q=1 | Time18mo | -0.072244681 | 0.399255202 | -0.180948627 | 0.8564079 |
|  | q=1 | Time24mo | -0.465033059 | 0.401477604 | -1.158303862 | 0.246740056 |
|  | q=2 | (Intercept) | 2.080047197 | 0.311647248 | 6.674364072 | 2.48E-11 |
|  | q=2 | Time18mo | -0.159164595 | 0.339898537 | -0.468270903 | 0.639590873 |
|  | q=2 | Time24mo | -0.52748909 | 0.34549669 | -1.526755841 | 0.126821736 |
|  | q=0 | (Intercept) | 5.44385809 | 0.292598262 | 18.60523041 | 2.91E-77 |
|  | q=0 | Bark_treatmentno bark | 0.009561423 | 0.316037044 | 0.030254121 | 0.975864386 |
|  | q=0 | Bark_treatmentwith bark | -0.07905098 | 0.318884825 | -0.247898218 | 0.804213159 |
|  | q=1 | (Intercept) | 2.838755787 | 0.364986393 | 7.777703059 | 7.39E-15 |
|  | q=1 | Bark_treatmentno bark | -0.07061444 | 0.394531114 | -0.178983198 | 0.857950895 |
|  | q=1 | Bark_treatmentwith bark | -0.49837131 | 0.400692315 | -1.243775565 | 0.213582176 |
|  | q=2 | (Intercept) | 2.080047143 | 0.314217508 | 6.619768443 | 3.60E-11 |
|  | q=2 | Bark_treatmentno bark | -0.18054293 | 0.34141015 | -0.528815355 | 0.596933545 |
|  | q=2 | Bark_treatmentwith bark | -0.520950777 | 0.350117368 | -1.487931832 | 0.136768872 |
| Non-bark effect group | q=0 | (Intercept) | 5.167851262 | 0.325369289 | 15.88303333 | 8.31E-57 |
|  | q=0 | Time18mo | -0.114600611 | 0.289965831 | -0.395221087 | 0.692679732 |
|  | q=0 | Time24mo | 0.126084769 | 0.312755389 | 0.403141795 | 0.686843915 |
|  | q=1 | (Intercept) | 2.257728948 | 0.523557856 | 4.312281679 | 1.62E-05 |
|  | q=1 | Time18mo | 0.046831689 | 0.565035989 | 0.082882666 | 0.933944837 |
|  | q=1 | Time24mo | 0.299344776 | 0.589504599 | 0.507790399 | 0.611600344 |
|  | q=2 | (Intercept) | 1.671683125 | 0.461604058 | 3.621465401 | 0.000292939 |
|  | q=2 | Time18mo | -0.037366362 | 0.516951928 | -0.072282083 | 0.942377423 |
|  | q=2 | Time24mo | 0.182803144 | 0.528081788 | 0.346164455 | 0.729219126 |
|  | q=0 | (Intercept) | 5.177276193 | 0.329185738 | 15.72752277 | 9.80E-56 |
|  | q=0 | Bark_treatmentno bark | -0.353869879 | 0.226088397 | -1.565183725 | 0.117539836 |
|  | q=0 | Bark_treatmentwith bark | 0.138441787 | 0.225142998 | 0.61490603 | 0.53861678 |
|  | q=1 | (Intercept) | 2.304125419 | 0.52328829 | 4.403166403 | 1.07E-05 |
|  | q=1 | Bark_treatmentno bark | -0.336618777 | 0.526796516 | -0.638992034 | 0.522828114 |
|  | q=1 | Bark_treatmentwith bark | 0.264487928 | 0.518884565 | 0.509724023 | 0.61024482 |
|  | q=2 | (Intercept) | 1.671698141 | 0.436847575 | 3.826730963 | 0.000129856 |
|  | q=2 | Bark_treatmentno bark | -0.189145737 | 0.500929012 | -0.377589904 | 0.705735266 |
|  | q=2 | Bark_treatmentwith bark | 0.264024648 | 0.488989829 | 0.539938936 | 0.589239145 |
| *Cunninghamia lanceolata* | q=0 | (Intercept) | 5.690359454 | 0.378501304 | 15.03392298 | 4.40E-51 |
|  | q=0 | Time18mo | 0.024692613 | 0.414607531 | 0.059556594 | 0.95250879 |
|  | q=0 | Time24mo | -0.513268545 | 0.409289768 | -1.254046852 | 0.209824976 |
|  | q=1 | (Intercept) | 2.933377598 | 0.529098273 | 5.544107305 | 2.95E-08 |
|  | q=1 | Time18mo | -0.135699429 | 0.580931032 | -0.233589568 | 0.815303627 |
|  | q=1 | Time24mo | -0.589141679 | 0.577684797 | -1.01983241 | 0.307807949 |
|  | q=2 | (Intercept) | 1.809803242 | 0.475170361 | 3.80874606 | 0.000139673 |
|  | q=2 | Time18mo | -0.057831824 | 0.522391921 | -0.110705817 | 0.911849633 |
|  | q=2 | Time24mo | -0.178546409 | 0.518411877 | -0.344410337 | 0.730537716 |
|  | q=0 | (Intercept) | 5.690359454 | 0.452682469 | 12.57031109 | 3.08E-36 |
|  | q=0 | Bark_treatmentno bark | -0.363292674 | 0.489205111 | -0.742618314 | 0.457712798 |
|  | q=0 | Bark_treatmentwith bark | -0.095648075 | 0.495957175 | -0.192855512 | 0.847072127 |
|  | q=1 | (Intercept) | 2.933377598 | 0.556345357 | 5.272583942 | 1.35E-07 |
|  | q=1 | Bark_treatmentno bark | -0.226559434 | 0.602795289 | -0.37584805 | 0.707029864 |
|  | q=1 | Bark_treatmentwith bark | -0.541039825 | 0.615681683 | -0.878765504 | 0.379528434 |
|  | q=2 | (Intercept) | 1.809803242 | 0.404649443 | 4.472521276 | 7.73E-06 |
|  | q=2 | Bark_treatmentno bark | 0.08654167 | 0.434476345 | 0.199186149 | 0.842117134 |
|  | q=2 | Bark_treatmentwith bark | -0.44767122 | 0.463653932 | -0.965528791 | 0.334280022 |
| *Toona ciliata* | q=0 | (Intercept) | 5.370638028 | 0.514558865 | 10.43736371 | 1.67E-25 |
|  | q=0 | Time18mo | -0.269755856 | 0.556002996 | -0.485169789 | 0.62755591 |
|  | q=0 | Time24mo | 0.411955627 | 0.563392316 | 0.731205619 | 0.464653569 |
|  | q=1 | (Intercept) | 3.004061406 | 0.444569647 | 6.757234609 | 1.41E-11 |
|  | q=1 | Time18mo | -0.69986413 | 0.488833448 | -1.431702625 | 0.152228945 |
|  | q=1 | Time24mo | -0.391896064 | 0.491862429 | -0.796759504 | 0.425590718 |
|  | q=2 | (Intercept) | 2.373172184 | 0.305266554 | 7.774098256 | 7.60E-15 |
|  | q=2 | Time18mo | -0.70386404 | 0.352965148 | -1.994146003 | **0.046136101** |
|  | q=2 | Time24mo | -0.622299582 | 0.357648914 | -1.739973358 | **0.081863696** |
|  | q=0 | (Intercept) | 5.370638028 | 0.575219532 | 9.336675362 | 9.94E-21 |
|  | q=0 | Bark_treatmentno bark | 0.286518605 | 0.62115267 | 0.461269216 | 0.644605468 |
|  | q=0 | Bark_treatmentwith bark | -0.188854478 | 0.63027484 | -0.299638294 | 0.764453071 |
|  | q=1 | (Intercept) | 3.004061406 | 0.448211326 | 6.702332653 | 2.05E-11 |
|  | q=1 | Bark_treatmentno bark | -0.425456057 | 0.488628733 | -0.870714365 | 0.383910133 |
|  | q=1 | Bark_treatmentwith bark | -0.718277014 | 0.501491644 | -1.432281119 | 0.152063384 |
|  | q=2 | (Intercept) | 2.373172184 | 0.305266783 | 7.774092426 | 7.60E-15 |
|  | q=2 | Bark_treatmentno bark | -0.650409313 | 0.350642766 | -1.854905835 | **0.063609675** |
|  | q=2 | Bark_treatmentwith bark | -0.684951934 | 0.360774281 | -1.898560873 | **0.057622237** |
| *Tectona grandis* | q=0 | (Intercept) | 5.209486153 | 0.466445628 | 11.16847461 | 5.82E-29 |
|  | q=0 | Time18mo | 0.31928134 | 0.503571754 | 0.634033458 | 0.526058987 |
|  | q=0 | Time24mo | -0.171451885 | 0.503987876 | -0.340190494 | 0.733713076 |
|  | q=1 | (Intercept) | 2.511642089 | 0.852986481 | 2.944527429 | 0.003234483 |
|  | q=1 | Time18mo | 0.550366402 | 0.918219316 | 0.599384474 | 0.548916528 |
|  | q=1 | Time24mo | -0.355251185 | 0.924455583 | -0.384281507 | 0.700769813 |
|  | q=2 | (Intercept) | 1.97045158 | 0.741452445 | 2.657556252 | 0.007870945 |
|  | q=2 | Time18mo | 0.25197065 | 0.797622984 | 0.315901942 | 0.752076938 |
|  | q=2 | Time24mo | -0.725325017 | 0.816167126 | -0.888696681 | 0.374166115 |
|  | q=0 | (Intercept) | 5.209486153 | 0.531290069 | 9.805352014 | 1.07E-22 |
|  | q=0 | Bark_treatmentno bark | 0.130452888 | 0.573761804 | 0.227364191 | 0.820140563 |
|  | q=0 | Bark_treatmentwith bark | 0.076252431 | 0.573800601 | 0.132890121 | 0.89428028 |
|  | q=1 | (Intercept) | 2.511642089 | 0.910652873 | 2.758067496 | 0.005814419 |
|  | q=1 | Bark_treatmentno bark | 0.465251103 | 0.981056961 | 0.474234546 | 0.635332661 |
|  | q=1 | Bark_treatmentwith bark | -0.171415393 | 0.984901982 | -0.174043099 | 0.861831591 |
|  | q=2 | (Intercept) | 1.97045158 | 0.858647043 | 2.294833012 | 0.021742696 |
|  | q=2 | Bark_treatmentno bark | 0.081909457 | 0.926459267 | 0.088411288 | 0.92954979 |
|  | q=2 | Bark_treatmentwith bark | -0.377799471 | 0.933176905 | -0.404853002 | 0.685585567 |
| *Kleinhovia hospita* | q=0 | (Intercept) | 5.099866428 | 0.356168816 | 14.31867755 | 1.67E-46 |
|  | q=0 | Time18mo | -0.431721443 | 0.438098611 | -0.985443531 | 0.324406256 |
|  | q=1 | (Intercept) | 2.594296587 | 0.783073955 | 3.312964975 | 0.000923125 |
|  | q=1 | Time18mo | -1.310466377 | 1.010418342 | -1.296954263 | 0.194646924 |
|  | q=2 | (Intercept) | 1.961643038 | 0.679614165 | 2.886406936 | 0.00389668 |
|  | q=2 | Time18mo | -0.989069939 | 0.900862902 | -1.097913941 | 0.27224207 |
|  | q=0 | (Intercept) | 5.099866428 | 0.080252348 | 63.54787818 | 0 |
|  | q=0 | Bark_treatmentno bark | -0.730418575 | 0.139432943 | -5.238493575 | **1.62E-07** |
|  | q=0 | Bark_treatmentwith bark | -0.202026628 | 0.119356626 | -1.692630178 | **0.090525883** |
|  | q=1 | (Intercept) | 2.594296587 | 0.274849565 | 9.438969217 | 3.76E-21 |
|  | q=1 | Bark_treatmentno bark | -2.338264503 | 0.922227357 | -2.535453418 | **0.011230186** |
|  | q=1 | Bark_treatmentwith bark | -0.814425863 | 0.495021328 | -1.645233885 | **0.099921588** |
|  | q=2 | (Intercept) | 1.961643038 | 0.376436028 | 5.211092695 | 1.88E-07 |
|  | q=2 | Bark_treatmentno bark | -1.897404951 | 1.039501174 | -1.825303327 | **0.067955268** |
|  | q=2 | Bark_treatmentwith bark | -0.521065169 | 0.616094049 | -0.845755887 | 0.397688939 |
| *Dipterocarpus turbinatus* | q=0 | (Intercept) | 5.225746674 | 0.364814554 | 14.32439198 | 1.54E-46 |
|  | q=0 | Time18mo | 0.059991911 | 0.393978544 | 0.152272025 | 0.878972389 |
|  | q=0 | Time24mo | 0.239496564 | 0.393802527 | 0.608164112 | 0.543078634 |
|  | q=1 | (Intercept) | 1.671016858 | 0.69537704 | 2.403037147 | 0.016259536 |
|  | q=1 | Time18mo | 0.878287554 | 0.738796865 | 1.188807906 | 0.234515266 |
|  | q=1 | Time24mo | 0.948037092 | 0.738202842 | 1.284250125 | 0.199054452 |
|  | q=2 | (Intercept) | 1.261642002 | 0.706158776 | 1.786626527 | 0.073997871 |
|  | q=2 | Time18mo | 0.522263664 | 0.75004596 | 0.696308882 | 0.486235412 |
|  | q=2 | Time24mo | 0.592852579 | 0.748772875 | 0.791765567 | 0.428497383 |
|  | q=0 | (Intercept) | 5.225746674 | 0.272203077 | 19.19797061 | 3.85E-82 |
|  | q=0 | Bark_treatmentno bark | -0.1198012 | 0.294206806 | -0.407200641 | 0.683860631 |
|  | q=0 | Bark_treatmentwith bark | 0.368344935 | 0.293543023 | 1.254824357 | 0.209542529 |
|  | q=1 | (Intercept) | 1.671016858 | 0.6213359 | 2.689393702 | 0.007158194 |
|  | q=1 | Bark_treatmentno bark | 0.595723304 | 0.660555559 | 0.901851928 | 0.367135531 |
|  | q=1 | Bark_treatmentwith bark | 1.154699901 | 0.654932379 | 1.76308263 | **0.077886554** |
|  | q=2 | (Intercept) | 1.261642002 | 0.671227804 | 1.87960331 | 0.060162162 |
|  | q=2 | Bark_treatmentno bark | 0.339941767 | 0.715566875 | 0.475066383 | 0.634739659 |
|  | q=2 | Bark_treatmentwith bark | 0.737219333 | 0.707828625 | 1.041522351 | 0.297633186 |

**Table S7.** Average relative abundance of fungal operational taxonomic units (OTUs) assigned to Phylum in bark effect and non-bark effect groups

|  |  | Variable | chi-squared | DF | p-value |
| --- | --- | --- | --- | --- | --- |
| Bark effect group | Ascomycota | (Intercept) | 2.5848 | 1 | 0.1079 |
|  |  | Time | 0.0105 | 1 | 0.9183 |
|  |  | Bark_treatment | 3.8835 | 1 | **0.0488 *** |
|  | Basidiomycota | (Intercept) | 3.5498 | 1 | 0.0596 |
|  |  | Time | 0.0524 | 1 | 0.819 |
|  |  | Bark_treatment | 4.0233 | 1 | **0.0449 *** |
| Non-bark effect group | Ascomycota | (Intercept) | 0.0151 | 1 | 0.9022 |
|  |  | Time | 0.5626 | 1 | 0.4532 |
|  |  | Bark_treatment | 0.1158 | 1 | 0.7336 |
|  | Basidiomycota | (Intercept) | 0.1029 | 1 | 0.7484 |
|  |  | Time | 0.3774 | 1 | 0.539 |
|  |  | Bark_treatment | 0.2457 | 1 | 0.6202 |

**Signif. codes :** 0 ‘***’ 0.001 ‘**’ 0.01 ‘*’ 0.05 ‘.’ 0.1 ‘ ’ 1

**Table S8.** Average relative abundance of fungal operational taxonomic units (OTUs) assigned to Decay types in bark effect and non-bark effect groups

|  |  | Variable | chi-squared | DF | p-value |
| --- | --- | --- | --- | --- | --- |
| Bark effect group | Soft rot | (Intercept) | 183.399 | 1 | 0 |
|  |  | Time | 2.6286 | 1 | 0.105 |
|  |  | Bark_treatment | 0.1932 | 1 | 0.6603 |
|  | White rot | (Intercept) | 115.7912 | 1 | 0 |
|  |  | Time | 1.021 | 1 | 0.3123 |
|  |  | Bark_treatment | 0.1982 | 1 | 0.6562 |
|  | Brown rot | (Intercept) | 798.7194 | 1 | 0 |
|  |  | Time | 0.0096 | 1 | 0.9218 |
|  |  | Bark_treatment | 0.0393 | 1 | 0.8428 |
| Non-bark effect group | Soft rot | (Intercept) | 46.6486 | 1 | 0 |
|  |  | Time | 0.1995 | 1 | 0.6552 |
|  |  | Bark_treatment | 0.4534 | 1 | 0.5007 |
|  | White rot | (Intercept) | 434.5903 | 1 | 0 |
|  |  | Time | 0.4253 | 1 | 0.5143 |
|  |  | Bark_treatment | 4.067 | 1 | **0.0437 *** |
|  | Brown rot | (Intercept) | 1.65133E+11 | 1 | 0 |
|  |  | Time | 0.0029 | 1 | 0.9568 |
|  |  | Bark_treatment | 0.0029 | 1 | 0.9569 |

**Signif. codes :** 0 ‘***’ 0.001 ‘**’ 0.01 ‘*’ 0.05 ‘.’ 0.1 ‘ ’ 1

**Table S9.** Average relative abundance of fungal operational taxonomic units (OTUs) assigned to Primary lifestyle in bark effect and non-bark effect groups

|  |  | Variable | chi-squared | DF | p-value |
| --- | --- | --- | --- | --- | --- |
| Bark effect group | Saprotroph | (Intercept) | 0.2834 | 1 | 0.5945 |
|  |  | Time | 0.2763 | 1 | 0.5991 |
|  |  | Bark_treatment | 0.0754 | 1 | 0.7837 |
|  | Plant pathogen | (Intercept) | 105.84 | 1 | 0 |
|  |  | Time | 7.5539 | 1 | **0.006 *** |
|  |  | Bark_treatment | 0.1073 | 1 | 0.7432 |
|  | Ectomycorrhizal | (Intercept) | 114.2845 | 1 | 0 |
|  |  | Time | 0.4924 | 1 | 0.4829 |
|  |  | Bark_treatment | 0.0106 | 1 | 0.9179 |
| Non-bark effect group | Saprotroph | (Intercept) | 0.0812 | 1 | 0.7756 |
|  |  | Time | 0.0115 | 1 | 0.9147 |
|  |  | Bark_treatment | 0.2645 | 1 | 0.6071 |
|  | Plant pathogen | (Intercept) | 49.5828 | 1 | 0 |
|  |  | Time | 0.2177 | 1 | 0.6408 |
|  |  | Bark_treatment | 0.0566 | 1 | 0.812 |
|  | Ectomycorrhizal | (Intercept) | 139.7502 | 1 | 0 |
|  |  | Time | 0.7922 | 1 | 0.3734 |
|  |  | Bark_treatment | 0.2162 | 1 | 0.6419 |

**Signif. codes :** 0 ‘***’ 0.001 ‘**’ 0.01 ‘*’ 0.05 ‘.’ 0.1 ‘ ’ 1

**Table S10.** Statistical analyses procedure summary for phospholipid fatty acid (PLFA) based on Generalized Additive Mixed Models (GAMM)

| Analytical approach | Response variable | Starting model | Final model (simplified and best model) |
| --- | --- | --- | --- |
| Generalized Additive Mixed Models (GAMM) | Fungi-Bacteria PLFA ratio | Fixed effects = Time + Bark treatment + Termites’ presence + Time: Bark treatment + Time:Termites’ presence + Bark treatment:Termites’ presence + Random effect = s(Species, bs = "re") | Fixed effects = Time + Bark treatment + Termites’ presence +  Random effect = s(Species, bs = "re") |
| Generalized Additive Mixed Models (GAMM) | Fungi PLFA | Fixed effects = Time + Bark treatment + Termites’ presence + Time: Bark treatment + Time:Termites’ presence + Bark treatment:Termites’ presence + Random effect = s(Species, bs = "re") | Fixed effects = Time + Bark treatment + Termites’ presence +  Random effect = s(Species, bs = "re") |
| Generalized Additive Mixed Models (GAMM) | Bacteria PLFA | Fixed effects = Time + Bark treatment + Termites’ presence + Time: Bark treatment + Time:Termites’ presence + Bark treatment:Termites’ presence + Random effect = s(Species, bs = "re") | Fixed effects = Time + Bark treatment + Termites’ presence +  Random effect = s(Species, bs = "re") |

**Table S11.** Model results for fungal and bacterial biomass ratio (Total phospholipid fatty acid, PLFA) dynamic across time and bark treatment in bark effect group

| Variable | Estimate | Standard error | z-value | p-value |
| --- | --- | --- | --- | --- |
| (Intercept) | 1.7542 | 0.1740 | 10.080 | **<2e-16** *** |
| Time.L | -0.3269 | 0.1624 | -2.013 | **0.0441 *** |
| Bark_treatmentbark | 0.1870 | 0.2175 | 0.860 | 0.3899 |
| TermitesP | 0.0265 | 0.4301 | 0.062 | 0.9509 |

**Table S12.** Model results for fungal biomass (phospholipid fatty acid, PLFA) dynamic across time and bark treatment in bark effect group

| Variable | Estimate | Standard error | z-value | p-value |
| --- | --- | --- | --- | --- |
| (Intercept) | 5.09206 | 0.55294 | 9.209 | **<2e-16 ***** |
| Time.L | -0.16039 | 0.18287 | -0.877 | 0.380 |
| Bark_treatmentbark | -0.02394 | 0.24783 | -0.097 | 0.923 |
| TermitesP | -0.74460 | 0.47923 | -1.554 | 0.120 |

**Table S13.** Model results for bacterial biomass (phospholipid fatty acid, PLFA) dynamic across of time and bark treatment in bark effect group

| Variable | Estimate | Standard error | z-value | p-value |
| --- | --- | --- | --- | --- |
| (Intercept) | 3.5128 | 0.4494 | 7.816 | **5.45e-15 ***** |
| Time.L | 0.3123 | 0.2045 | 1.527 | 0.127 |
| Bark_treatmentbark | -0.1700 | 0.2781 | -0.611 | 0.541 |
| TermitesP | -0.8784 | 0.5452 | -1.611 | 0.107 |

**Table S14.** Model results for fungal and bacterial biomass ratio (Total phospholipid fatty acid, PLFA) dynamic across time and bark treatment in non-bark effect group

| Variable | Estimate | Standard error | z-value | p-value |
| --- | --- | --- | --- | --- |
| (Intercept) | 1.37343 | 0.30859 | 4.451 | **8.56e-06 ***** |
| Time.L | -0.94409 | 0.26545 | -3.557 | **0.000376 ***** |
| Bark_treatmentbark | 0.44505 | 0.37171 | 1.197 | 0.231182 |
| TermitesP | -0.02878 | 0.56560 | -0.051 | 0.959424 |

**Table S15.** Model results for fungal biomass (phospholipid fatty acid, PLFA) dynamic across time and bark treatment in non-bark effect group

| Variable | Estimate | Standard error | z-value | p-value |
| --- | --- | --- | --- | --- |
| (Intercept) | 6.6850 | 0.6673 | 10.017 | **<2e-16 ***** |
| Time.L | -0.5564 | 0.2568 | -2.167 | **0.0303 *** |
| Bark_treatmentbark | -0.1150 | 0.3706 | -0.310 | 0.7562 |
| TermitesP | -0.5850 | 0.5867 | -0.997 | 0.3187 |

**Table S16.** Model results for bacterial biomass (phospholipid fatty acid, PLFA) dynamic across time and bark treatment in non-bark effect group

| Variable | Estimate | Standard error | z-value | p-value |
| --- | --- | --- | --- | --- |
| (Intercept) | 5.4818 | 0.8127 | 6.745 | **1.53e-11 ***** |
| Time.L | 0.4372 | 0.2333 | 1.874 | **0.061 .** |
| Bark_treatmentbark | -0.3282 | 0.3343 | -0.982 | 0.326 |
| TermitesP | -0.7926 | 0.5347 | -1.482 | 0.138 |

**Signif. codes :** 0 ‘***’ 0.001 ‘**’ 0.01 ‘*’ 0.05 ‘.’ 0.1 ‘ ’ 1

**Table S17.** Changes in fungal beta diversity (turnover and nestedness) across bark treatments over time for bark effect and non-bark effect groups

| Bark effect group | group | beta.sim | beta.sne |
| --- | --- | --- | --- |
|  | 0mo_initial | 0.022759623 | 0.083526156 |
|  | 18mo_no bark | 0.489349636 | 0.109935141 |
|  | 18mo_with bark | 0.063724209 | 0.068945916 |
|  | 24mo_no bark | 0.391765113 | 0.262637258 |
|  | 24mo_with bark | 0.358635327 | 0.287037156 |
| Non-bark effect group | 0mo_initial | 0.958139535 | 0.032077856 |
|  | 18mo_no bark | 0.558184127 | 0.27553778 |
|  | 18mo_with bark | 0.48175343 | 0.214056958 |
|  | 24mo_no bark | 0.747343302 | 0.06855534 |
|  | 24mo_with bark | 0.112288786 | 0.609797599 |

**Table S18.** Correlations between fungal community composition and wood physicochemical properties in bark effect and non-bark effect groups

| **Group** | **Phylum/Decay type/Primary lifestyle** | **Physicochemical property** | **Mantel's r** | **P-value** | **Mantel's r** | **P-value**  **(FDR adj)** |
| --- | --- | --- | --- | --- | --- | --- |
| Combined group | Ascomycota | C | – | – | 0.076 | 0.138 |
| Combined group | Ascomycota | N | – | – | 0.177 | 0.008 * |
| Combined group | Ascomycota | P | – | – | 0.132 | 0.023 * |
| Combined group | Ascomycota | K | – | – | 0.071 | 0.117 |
| Combined group | Ascomycota | Ca | – | – | 0.096 | 0.075 |
| Combined group | Ascomycota | Fe | – | – | 0.009 | 0.560 |
| Combined group | Ascomycota | Mn | – | – | -0.001 | 0.611 |
| Combined group | Ascomycota | Cu | – | – | 0.050 | 0.165 |
| Combined group | Ascomycota | Zn | – | – | 0.008 | 0.550 |
| Combined group | Ascomycota | Lignin | – | – | 0.085 | 0.073 |
| Combined group | Ascomycota | Sugar | – | – | 0.031 | 0.382 |
| Combined group | Ascomycota | Tannins | – | – | 0.077 | 0.117 |
| Combined group | Ascomycota | Fiber content | – | – | 0.081 | 0.073 |
| Combined group | Ascomycota | Hemicellulose | – | – | 0.061 | 0.168 |
| Combined group | Ascomycota | Cellulose | – | – | 0.052 | 0.200 |
| Combined group | Ascomycota | Water content | – | – | 0.076 | 0.117 |
| Combined group | Basidiomycota | C | – | – | 0.081 | 0.116 |
| Combined group | Basidiomycota | N | – | – | 0.197 | 0.008 * |
| Combined group | Basidiomycota | P | – | – | 0.138 | 0.018 * |
| Combined group | Basidiomycota | K | – | – | 0.074 | 0.117 |
| Combined group | Basidiomycota | Ca | – | – | 0.097 | 0.077 |
| Combined group | Basidiomycota | Fe | – | – | 0.007 | 0.559 |
| Combined group | Basidiomycota | Mn | – | – | -0.001 | 0.605 |
| Combined group | Basidiomycota | Cu | – | – | 0.054 | 0.147 |
| Combined group | Basidiomycota | Zn | – | – | 0.009 | 0.521 |
| Combined group | Basidiomycota | Lignin | – | – | 0.092 | 0.070 |
| Combined group | Basidiomycota | Sugar | – | – | 0.043 | 0.301 |
| Combined group | Basidiomycota | Tannins | – | – | 0.112 | 0.070 |
| Combined group | Basidiomycota | Fiber content | – | – | 0.089 | 0.075 |
| Combined group | Basidiomycota | Hemicellulose | – | – | 0.065 | 0.187 |
| Combined group | Basidiomycota | Cellulose | – | – | 0.056 | 0.166 |
| Combined group | Basidiomycota | Water content | – | – | 0.083 | 0.109 |
| Combined group | Saprotroph | C | – | – | -0.002 | 0.624 |
| Combined group | Saprotroph | N | – | – | 0.042 | 0.341 |
| Combined group | Saprotroph | P | – | – | 0.023 | 0.444 |
| Combined group | Saprotroph | K | – | – | 0.013 | 0.510 |
| Combined group | Saprotroph | Ca | – | – | 0.099 | 0.079 |
| Combined group | Saprotroph | Fe | – | – | -0.037 | 0.899 |
| Combined group | Saprotroph | Mn | – | – | -0.046 | 0.921 |
| Combined group | Saprotroph | Cu | – | – | 0.026 | 0.394 |
| Combined group | Saprotroph | Zn | – | – | -0.042 | 0.914 |
| Combined group | Saprotroph | Lignin | – | – | 0.035 | 0.377 |
| Combined group | Saprotroph | Sugar | – | – | 0.083 | 0.117 |
| Combined group | Saprotroph | Tannins | – | – | -0.015 | 0.708 |
| Combined group | Saprotroph | Fiber content | – | – | 0.041 | 0.345 |
| Combined group | Saprotroph | Hemicellulose | – | – | 0.002 | 0.616 |
| Combined group | Saprotroph | Cellulose | – | – | 0.020 | 0.481 |
| Combined group | Saprotroph | Water content | – | – | 0.151 | 0.032 * |
| Combined group | Plant pathogen | C | – | – | -0.027 | 0.713 |
| Combined group | Plant pathogen | N | – | – | 0.031 | 0.493 |
| Combined group | Plant pathogen | P | – | – | 0.162 | 0.084 |
| Combined group | Plant pathogen | K | – | – | 0.204 | 0.043 * |
| Combined group | Plant pathogen | Ca | – | – | 0.075 | 0.345 |
| Combined group | Plant pathogen | Fe | – | – | 0.023 | 0.510 |
| Combined group | Plant pathogen | Mn | – | – | -0.050 | 0.856 |
| Combined group | Plant pathogen | Cu | – | – | 0.154 | 0.028 * |
| Combined group | Plant pathogen | Zn | – | – | 0.030 | 0.493 |
| Combined group | Plant pathogen | Lignin | – | – | 0.278 | 0.008 * |
| Combined group | Plant pathogen | Sugar | – | – | 0.206 | 0.046 * |
| Combined group | Plant pathogen | Tannins | – | – | 0.199 | 0.072 |
| Combined group | Plant pathogen | Fiber content | – | – | 0.148 | 0.075 |
| Combined group | Plant pathogen | Hemicellulose | – | – | -0.075 | 0.899 |
| Combined group | Plant pathogen | Cellulose | – | – | 0.113 | 0.190 |
| Combined group | Plant pathogen | Water content | – | – | 0.068 | 0.357 |
| Combined group | Ectomycorrhizal | C | – | – | -0.083 | 0.926 |
| Combined group | Ectomycorrhizal | N | – | – | 0.043 | 0.420 |
| Combined group | Ectomycorrhizal | P | – | – | -0.036 | 0.781 |
| Combined group | Ectomycorrhizal | K | – | – | -0.081 | 0.926 |
| Combined group | Ectomycorrhizal | Ca | – | – | -0.097 | 0.934 |
| Combined group | Ectomycorrhizal | Fe | – | – | -0.090 | 0.934 |
| Combined group | Ectomycorrhizal | Mn | – | – | -0.015 | 0.691 |
| Combined group | Ectomycorrhizal | Cu | – | – | -0.088 | 0.983 |
| Combined group | Ectomycorrhizal | Zn | – | – | -0.092 | 0.934 |
| Combined group | Ectomycorrhizal | Lignin | – | – | -0.086 | 0.934 |
| Combined group | Ectomycorrhizal | Sugar | – | – | 0.051 | 0.439 |
| Combined group | Ectomycorrhizal | Tannins | – | – | 0.132 | 0.200 |
| Combined group | Ectomycorrhizal | Fiber content | – | – | -0.114 | 0.973 |
| Combined group | Ectomycorrhizal | Hemicellulose | – | – | -0.093 | 0.927 |
| Combined group | Ectomycorrhizal | Cellulose | – | – | -0.099 | 0.934 |
| Combined group | Ectomycorrhizal | Water content | – | – | -0.034 | 0.758 |
| Combined group | Soft rot | C | – | – | 0.034 | 0.500 |
| Combined group | Soft rot | N | – | – | -0.066 | 0.899 |
| Combined group | Soft rot | P | – | – | 0.118 | 0.162 |
| Combined group | Soft rot | K | – | – | 0.106 | 0.231 |
| Combined group | Soft rot | Ca | – | – | -0.022 | 0.708 |
| Combined group | Soft rot | Fe | – | – | 0.113 | 0.190 |
| Combined group | Soft rot | Mn | – | – | 0.007 | 0.602 |
| Combined group | Soft rot | Cu | – | – | 0.001 | 0.607 |
| Combined group | Soft rot | Zn | – | – | 0.061 | 0.357 |
| Combined group | Soft rot | Lignin | – | – | -0.033 | 0.775 |
| Combined group | Soft rot | Sugar | – | – | 0.007 | 0.577 |
| Combined group | Soft rot | Tannins | – | – | -0.092 | 0.927 |
| Combined group | Soft rot | Fiber content | – | – | 0.045 | 0.406 |
| Combined group | Soft rot | Hemicellulose | – | – | 0.007 | 0.602 |
| Combined group | Soft rot | Cellulose | – | – | 0.083 | 0.256 |
| Combined group | Soft rot | Water content | – | – | 0.022 | 0.526 |
| Combined group | White rot | C | – | – | -0.044 | 0.784 |
| Combined group | White rot | N | – | – | -0.026 | 0.750 |
| Combined group | White rot | P | – | – | 0.135 | 0.147 |
| Combined group | White rot | K | – | – | 0.091 | 0.293 |
| Combined group | White rot | Ca | – | – | -0.007 | 0.654 |
| Combined group | White rot | Fe | – | – | -0.021 | 0.708 |
| Combined group | White rot | Mn | – | – | -0.086 | 0.927 |
| Combined group | White rot | Cu | – | – | 0.145 | 0.048 * |
| Combined group | White rot | Zn | – | – | -0.034 | 0.758 |
| Combined group | White rot | Lignin | – | – | 0.215 | 0.035 * |
| Combined group | White rot | Sugar | – | – | 0.153 | 0.129 |
| Combined group | White rot | Tannins | – | – | 0.225 | 0.048 * |
| Combined group | White rot | Fiber content | – | – | 0.093 | 0.251 |
| Combined group | White rot | Hemicellulose | – | – | -0.162 | 0.995 |
| Combined group | White rot | Cellulose | – | – | 0.075 | 0.347 |
| Combined group | White rot | Water content | – | – | -0.095 | 0.927 |
| Combined group | Brown rot | C | – | – | -0.040 | 0.758 |
| Combined group | Brown rot | N | – | – | 0.085 | 0.301 |
| Combined group | Brown rot | P | – | – | -0.019 | 0.696 |
| Combined group | Brown rot | K | – | – | -0.133 | 0.973 |
| Combined group | Brown rot | Ca | – | – | -0.189 | 0.995 |
| Combined group | Brown rot | Fe | – | – | -0.071 | 0.886 |
| Combined group | Brown rot | Mn | – | – | -0.043 | 0.775 |
| Combined group | Brown rot | Cu | – | – | -0.049 | 0.899 |
| Combined group | Brown rot | Zn | – | – | -0.124 | 0.963 |
| Combined group | Brown rot | Lignin | – | – | 0.036 | 0.489 |
| Combined group | Brown rot | Sugar | – | – | 0.075 | 0.357 |
| Combined group | Brown rot | Tannins | – | – | 0.383 | 0.013 * |
| Combined group | Brown rot | Fiber content | – | – | -0.078 | 0.914 |
| Combined group | Brown rot | Hemicellulose | – | – | -0.115 | 0.934 |
| Combined group | Brown rot | Cellulose | – | – | -0.060 | 0.847 |
| Combined group | Brown rot | Water content | – | – | -0.017 | 0.708 |
| Combined group | Mass loss percentage | C | – | – | 0.232 | 0.008 * |
| Combined group | Mass loss percentage | N | – | – | 0.146 | 0.013 * |
| Combined group | Mass loss percentage | P | – | – | 0.133 | 0.028 * |
| Combined group | Mass loss percentage | K | – | – | 0.127 | 0.028 * |
| Combined group | Mass loss percentage | Ca | – | – | 0.078 | 0.117 |
| Combined group | Mass loss percentage | Fe | – | – | 0.275 | 0.008 * |
| Combined group | Mass loss percentage | Mn | – | – | 0.310 | 0.008 * |
| Combined group | Mass loss percentage | Cu | – | – | 0.290 | 0.008 * |
| Combined group | Mass loss percentage | Zn | – | – | 0.266 | 0.008 * |
| Combined group | Mass loss percentage | Lignin | – | – | 0.318 | 0.008 * |
| Combined group | Mass loss percentage | Sugar | – | – | 0.075 | 0.132 |
| Combined group | Mass loss percentage | Tannins | – | – | 0.066 | 0.194 |
| Combined group | Mass loss percentage | Fiber content | – | – | 0.335 | 0.008 * |
| Combined group | Mass loss percentage | Hemicellulose | – | – | 0.089 | 0.094 |
| Combined group | Mass loss percentage | Cellulose | – | – | 0.203 | 0.008 * |
| Combined group | Mass loss percentage | Water content | – | – | 0.169 | 0.008 * |
| Combined group | Total (fungal and bacterial) biomass | C | – | – | -0.0005 | 0.624 |
| Combined group | Total (fungal and bacterial) biomass | N | – | – | 0.004 | 0.611 |
| Combined group | Total (fungal and bacterial) biomass | P | – | – | 0.059 | 0.329 |
| Combined group | Total (fungal and bacterial) biomass | K | – | – | 0.207 | 0.025 * |
| Combined group | Total (fungal and bacterial) biomass | Ca | – | – | 0.050 | 0.382 |
| Combined group | Total (fungal and bacterial) biomass | Fe | – | – | 0.089 | 0.194 |
| Combined group | Total (fungal and bacterial) biomass | Mn | – | – | 0.039 | 0.406 |
| Combined group | Total (fungal and bacterial) biomass | Cu | – | – | 0.063 | 0.213 |
| Combined group | Total (fungal and bacterial) biomass | Zn | – | – | 0.064 | 0.319 |
| Combined group | Total (fungal and bacterial) biomass | Lignin | – | – | 0.081 | 0.200 |
| Combined group | Total (fungal and bacterial) biomass | Sugar | – | – | 0.142 | 0.070 |
| Combined group | Total (fungal and bacterial) biomass | Tannins | – | – | 0.015 | 0.602 |
| Combined group | Total (fungal and bacterial) biomass | Fiber content | – | – | 0.048 | 0.382 |
| Combined group | Total (fungal and bacterial) biomass | Hemicellulose | – | – | 0.005 | 0.602 |
| Combined group | Total (fungal and bacterial) biomass | Cellulose | – | – | 0.047 | 0.406 |
| Combined group | Total (fungal and bacterial) biomass | Water content | – | – | 0.099 | 0.196 |
| Combined group | Fungal biomass | C | – | – | 0.545 | 0.008 * |
| Combined group | Fungal biomass | N | – | – | 0.385 | 0.008 * |
| Combined group | Fungal biomass | P | – | – | 0.282 | 0.008 * |
| Combined group | Fungal biomass | K | – | – | 0.276 | 0.013 * |
| Combined group | Fungal biomass | Ca | – | – | 0.233 | 0.018 * |
| Combined group | Fungal biomass | Fe | – | – | 0.385 | 0.008 * |
| Combined group | Fungal biomass | Mn | – | – | 0.248 | 0.008 * |
| Combined group | Fungal biomass | Cu | – | – | 0.240 | 0.008 * |
| Combined group | Fungal biomass | Zn | – | – | 0.443 | 0.008 * |
| Combined group | Fungal biomass | Lignin | – | – | 0.188 | 0.025 * |
| Combined group | Fungal biomass | Sugar | – | – | 0.016 | 0.573 |
| Combined group | Fungal biomass | Tannins | – | – | -0.005 | 0.624 |
| Combined group | Fungal biomass | Fiber content | – | – | 0.214 | 0.025 * |
| Combined group | Fungal biomass | Hemicellulose | – | – | 0.488 | 0.008 * |
| Combined group | Fungal biomass | Cellulose | – | – | 0.144 | 0.105 |
| Combined group | Fungal biomass | Water content | – | – | 0.035 | 0.485 |
| Combined group | Bacterial biomass | C | – | – | 0.354 | 0.008 * |
| Combined group | Bacterial biomass | N | – | – | 0.312 | 0.008 * |
| Combined group | Bacterial biomass | P | – | – | 0.165 | 0.059 |
| Combined group | Bacterial biomass | K | – | – | 0.102 | 0.198 |
| Combined group | Bacterial biomass | Ca | – | – | 0.174 | 0.075 |
| Combined group | Bacterial biomass | Fe | – | – | 0.221 | 0.023 * |
| Combined group | Bacterial biomass | Mn | – | – | 0.298 | 0.008 * |
| Combined group | Bacterial biomass | Cu | – | – | 0.201 | 0.013 * |
| Combined group | Bacterial biomass | Zn | – | – | 0.300 | 0.008 * |
| Combined group | Bacterial biomass | Lignin | – | – | 0.204 | 0.025 * |
| Combined group | Bacterial biomass | Sugar | – | – | 0.041 | 0.453 |
| Combined group | Bacterial biomass | Tannins | – | – | -0.037 | 0.781 |
| Combined group | Bacterial biomass | Fiber content | – | – | 0.213 | 0.013 * |
| Combined group | Bacterial biomass | Hemicellulose | – | – | 0.248 | 0.018 * |
| Combined group | Bacterial biomass | Cellulose | – | – | 0.112 | 0.194 |
| Combined group | Bacterial biomass | Water content | – | – | 0.221 | 0.035 * |
| Bark effect group | Ascomycota | C | 0.041 | 0.21 | 0.041 | 0.393 |
| Bark effect group | Ascomycota | N | 0.112 | 0.032* | 0.112 | 0.208 |
| Bark effect group | Ascomycota | P | 0.051 | 0.160 | 0.051 | 0.353 |
| Bark effect group | Ascomycota | K | 0.061 | 0.159 | 0.061 | 0.353 |
| Bark effect group | Ascomycota | Ca | 0.086 | 0.077 | 0.086 | 0.255 |
| Bark effect group | Ascomycota | Fe | 0.017 | 0.348 | 0.017 | 0.543 |
| Bark effect group | Ascomycota | Mn | 0.081 | 0.065 | 0.081 | 0.231 |
| Bark effect group | Ascomycota | Cu | 0.026 | 0.262 | 0.026 | 0.466 |
| Bark effect group | Ascomycota | Zn | 0.026 | 0.292 | 0.027 | 0.498 |
| Bark effect group | Ascomycota | Lignin | 0.019 | 0.323 | 0.019 | 0.521 |
| Bark effect group | Ascomycota | Sugar | -0.0003 | 0.444 | -0.0003 | 0.610 |
| Bark effect group | Ascomycota | Tannins | 0.019 | 0.323 | 0.019 | 0.521 |
| Bark effect group | Ascomycota | Fiber content | -0.001 | 0.459 | -0.001 | 0.610 |
| Bark effect group | Ascomycota | Hemicellulose | -0.056 | 0.863 | -0.056 | 0.905 |
| Bark effect group | Ascomycota | Cellulose | 0.0003 | 0.451 | 0.0003 | 0.610 |
| Bark effect group | Ascomycota | Water content | 0.102 | 0.046* | 0.102 | 0.226 |
| Bark effect group | Basidiomycota | C | 0.036 | 0.221 | 0.036 | 0.408 |
| Bark effect group | Basidiomycota | N | 0.134 | 0.013 | 0.135 | 0.139 |
| Bark effect group | Basidiomycota | P | 0.061 | 0.126 | 0.061 | 0.330 |
| Bark effect group | Basidiomycota | K | 0.068 | 0.134 | 0.068 | 0.332 |
| Bark effect group | Basidiomycota | Ca | 0.087 | 0.073 | 0.087 | 0.246 |
| Bark effect group | Basidiomycota | Fe | 0.012 | 0.384 | 0.013 | 0.567 |
| Bark effect group | Basidiomycota | Mn | 0.087 | 0.054 | 0.087 | 0.230 |
| Bark effect group | Basidiomycota | Cu | 0.028 | 0.242 | 0.028 | 0.434 |
| Bark effect group | Basidiomycota | Zn | 0.016 | 0.366 | 0.016 | 0.555 |
| Bark effect group | Basidiomycota | Lignin | 0.027 | 0.291 | 0.028 | 0.498 |
| Bark effect group | Basidiomycota | Sugar | 0.018 | 0.335 | 0.018 | 0.536 |
| Bark effect group | Basidiomycota | Tannins | 0.060 | 0.134 | 0.060 | 0.332 |
| Bark effect group | Basidiomycota | Fiber content | -0.002 | 0.476 | -0.002 | 0.622 |
| Bark effect group | Basidiomycota | Hemicellulose | -0.054 | 0.837 | -0.054 | 0.891 |
| Bark effect group | Basidiomycota | Cellulose | 0.003 | 0.440 | 0.003 | 0.610 |
| Bark effect group | Basidiomycota | Water content | 0.131 | 0.020* | 0.131 | 0.175 |
| Bark effect group | Saprotroph | C | -0.029 | 0.688 | -0.029 | 0.805 |
| Bark effect group | Saprotroph | N | 0.047 | 0.189 | 0.047 | 0.371 |
| Bark effect group | Saprotroph | P | 0.008 | 0.367 | 0.009 | 0.555 |
| Bark effect group | Saprotroph | K | 0.040 | 0.234 | 0.041 | 0.424 |
| Bark effect group | Saprotroph | Ca | 0.078 | 0.083 | 0.079 | 0.270 |
| Bark effect group | Saprotroph | Fe | 0.059 | 0.152 | 0.059 | 0.347 |
| Bark effect group | Saprotroph | Mn | 0.089 | 0.063 | 0.090 | 0.231 |
| Bark effect group | Saprotroph | Cu | 0.018 | 0.283 | 0.019 | 0.498 |
| Bark effect group | Saprotroph | Zn | 0.018 | 0.315 | 0.018 | 0.517 |
| Bark effect group | Saprotroph | Lignin | -0.045 | 0.809 | -0.046 | 0.874 |
| Bark effect group | Saprotroph | Sugar | 0.064 | 0.141 | 0.064 | 0.338 |
| Bark effect group | Saprotroph | Tannins | -0.058 | 0.861 | -0.058 | 0.905 |
| Bark effect group | Saprotroph | Fiber content | -0.037 | 0.794 | -0.038 | 0.871 |
| Bark effect group | Saprotroph | Hemicellulose | -0.089 | 0.967 | -0.089 | 0.993 |
| Bark effect group | Saprotroph | Cellulose | -0.086 | 0.980 | -0.087 | 0.995 |
| Bark effect group | Saprotroph | Water content | 0.253 | 0.001* | 0.253 | 0.048 * |
| Bark effect group | Plant pathogen | C | 0.069 | 0.179 | 0.070 | 0.371 |
| Bark effect group | Plant pathogen | N | 0.052 | 0.224 | 0.052 | 0.410 |
| Bark effect group | Plant pathogen | P | 0.156 | 0.026* | 0.156 | 0.207 |
| Bark effect group | Plant pathogen | K | 0.209 | 0.017* | 0.210 | 0.155 |
| Bark effect group | Plant pathogen | Ca | 0.164 | 0.035* | 0.164 | 0.208 |
| Bark effect group | Plant pathogen | Fe | 0.08 | 0.137 | 0.086 | 0.333 |
| Bark effect group | Plant pathogen | Mn | 0.056 | 0.190 | 0.056 | 0.371 |
| Bark effect group | Plant pathogen | Cu | 0.129 | 0.041* | 0.129 | 0.219 |
| Bark effect group | Plant pathogen | Zn | 0.139 | 0.065 | 0.139 | 0.231 |
| Bark effect group | Plant pathogen | Lignin | 0.150 | 0.032* | 0.150 | 0.208 |
| Bark effect group | Plant pathogen | Sugar | 0.169 | 0.030* | 0.170 | 0.208 |
| Bark effect group | Plant pathogen | Tannins | 0.184 | 0.028* | 0.185 | 0.207 |
| Bark effect group | Plant pathogen | Fiber content | 0.088 | 0.060 | 0.088 | 0.231 |
| Bark effect group | Plant pathogen | Hemicellulose | -0.002 | 0.461 | -0.002 | 0.610 |
| Bark effect group | Plant pathogen | Cellulose | 0.134 | 0.045* | 0.134 | 0.226 |
| Bark effect group | Plant pathogen | Water content | 0.072 | 0.171 | 0.072 | 0.371 |
| Bark effect group | Ectomycorrhizal | C | -0.045 | 0.612 | -0.045 | 0.754 |
| Bark effect group | Ectomycorrhizal | N | 0.089 | 0.147 | 0.089 | 0.344 |
| Bark effect group | Ectomycorrhizal | P | -0.010 | 0.480 | -0.011 | 0.623 |
| Bark effect group | Ectomycorrhizal | K | -0.076 | 0.715 | -0.076 | 0.822 |
| Bark effect group | Ectomycorrhizal | Ca | -0.054 | 0.617 | -0.054 | 0.800 |
| Bark effect group | Ectomycorrhizal | Fe | -0.088 | 0.831 | -0.088 | 0.891 |
| Bark effect group | Ectomycorrhizal | Mn | 0.012 | 0.401 | 0.012 | 0.579 |
| Bark effect group | Ectomycorrhizal | Cu | -0.115 | 0.965 | -0.115 | 0.993 |
| Bark effect group | Ectomycorrhizal | Zn | -0.083 | 0.786 | -0.083 | 0.871 |
| Bark effect group | Ectomycorrhizal | Lignin | -0.047 | 0.676 | -0.047 | 0.800 |
| Bark effect group | Ectomycorrhizal | Sugar | 0.139 | 0.085 | 0.139 | 0.271 |
| Bark effect group | Ectomycorrhizal | Tannins | 0.171 | 0.065 | 0.171 | 0.231 |
| Bark effect group | Ectomycorrhizal | Fiber content | -0.030 | 0.618 | -0.030 | 0.756 |
| Bark effect group | Ectomycorrhizal | Hemicellulose | -0.080 | 0.780 | -0.080 | 0.871 |
| Bark effect group | Ectomycorrhizal | Cellulose | -0.05158 | 0.715 | -0.035 | 0.773 |
| Bark effect group | Ectomycorrhizal | Water content | 0.004 | 0.434 | 0.004 | 0.610 |
| Bark effect group | Soft rot | C | 0.001 | 0.457 | 0.001 | 0.610 |
| Bark effect group | Soft rot | N | -0.027 | 0.562 | -0.027 | 0.719 |
| Bark effect group | Soft rot | P | 0.187 | 0.034* | 0.187 | 0.208 |
| Bark effect group | Soft rot | K | 0.168 | 0.062 | 0.168 | 0.231 |
| Bark effect group | Soft rot | Ca | 0.066 | 0.193 | 0.066 | 0.371 |
| Bark effect group | Soft rot | Fe | 0.043 | 0.283 | 0.043 | 0.496 |
| Bark effect group | Soft rot | Mn | -0.018 | 0.549 | -0.018 | 0.707 |
| Bark effect group | Soft rot | Cu | 0.015 | 0.391 | 0.015 | 0.573 |
| Bark effect group | Soft rot | Zn | 0.032 | 0.347 | 0.032 | 0.543 |
| Bark effect group | Soft rot | Lignin | -0.063 | 0.792 | -0.063 | 0.871 |
| Bark effect group | Soft rot | Sugar | 0.078 | 0.183 | 0.078 | 0.371 |
| Bark effect group | Soft rot | Tannins | -0.043 | 0.643 | -0.043 | 0.776 |
| Bark effect group | Soft rot | Fiber content | 0.037 | 0.284 | 0.037 | 0.496 |
| Bark effect group | Soft rot | Hemicellulose | -0.040 | 0.613 | -0.040 | 0.754 |
| Bark effect group | Soft rot | Cellulose | 0.176 | 0.037* | 0.176 | 0.208 |
| Bark effect group | Soft rot | Water content | 0.080 | 0.184 | 0.080 | 0.371 |
| Bark effect group | White rot | C | 0.098 | 0.156 | 0.098 | 0.352 |
| Bark effect group | White rot | N | -0.004 | 0.470 | -0.004 | 0.618 |
| Bark effect group | White rot | P | 0.159 | 0.056* | 0.159 | 0.231 |
| Bark effect group | White rot | K | 0.143 | 0.097 | 0.143 | 0.287 |
| Bark effect group | White rot | Ca | 0.034 | 0.314 | 0.034 | 0.517 |
| Bark effect group | White rot | Fe | 0.114 | 0.125 | 0.114 | 0.330 |
| Bark effect group | White rot | Mn | 0.064 | 0.201 | 0.064 | 0.382 |
| Bark effect group | White rot | Cu | 0.094 | 0.126 | 0.094 | 0.330 |
| Bark effect group | White rot | Zn | 0.077 | 0.211 | 0.077 | 0.393 |
| Bark effect group | White rot | Lignin | 0.152 | 0.051* | 0.152 | 0.228 |
| Bark effect group | White rot | Sugar | 0.133 | 0.113 | 0.133 | 0.317 |
| Bark effect group | White rot | Tannins | 0.155 | 0.095 | 0.155 | 0.285 |
| Bark effect group | White rot | Fiber content | 0.089 | 0.118 | 0.089 | 0.324 |
| Bark effect group | White rot | Hemicellulose | -0.075 | 0.761 | -0.075 | 0.859 |
| Bark effect group | White rot | Cellulose | 0.081 | 0.192 | 0.081 | 0.371 |
| Bark effect group | White rot | Water content | -0.106 | 0.840 | -0.106 | 0.891 |
| Bark effect group | Brown rot | C | -0.045 | 0.585 | -0.045 | 0.744 |
| Bark effect group | Brown rot | N | 0.011 | 0.378 | 0.011 | 0.563 |
| Bark effect group | Brown rot | P | -0.068 | 0.714 | -0.068 | 0.822 |
| Bark effect group | Brown rot | K | -0.213 | 0.998 | -0.213 | 0.998 |
| Bark effect group | Brown rot | Ca | -0.177 | 0.988 | -0.177 | 0.995 |
| Bark effect group | Brown rot | Fe | -0.065 | 0.695 | -0.065 | 0.809 |
| Bark effect group | Brown rot | Mn | -0.063 | 0.733 | -0.063 | 0.833 |
| Bark effect group | Brown rot | Cu | -0.095 | 0.888 | -0.095 | 0.927 |
| Bark effect group | Brown rot | Zn | -0.182 | 0.990 | -0.182 | 0.995 |
| Bark effect group | Brown rot | Lignin | 0.027 | 0.359 | 0.027 | 0.555 |
| Bark effect group | Brown rot | Sugar | 0.143 | 0.114 | 0.143 | 0.317 |
| Bark effect group | Brown rot | Tannins | 0.412 | 0.003* | 0.412 | 0.064 |
| Bark effect group | Brown rot | Fiber content | -0.102 | 0.933 | -0.102 | 0.968 |
| Bark effect group | Brown rot | Hemicellulose | -0.175 | 0.984 | -0.175 | 0.995 |
| Bark effect group | Brown rot | Cellulose | 0.008 | 0.444 | 0.008 | 0.610 |
| Bark effect group | Brown rot | Water content | -0.106 | 0.810 | -0.106 | 0.874 |
| Bark effect group | Mass loss percentage | C | 0.068 | 0.174 | 0.068 | 0.371 |
| Bark effect group | Mass loss percentage | N | 0.090 | 0.095 | 0.090 | 0.285 |
| Bark effect group | Mass loss percentage | P | 0.133 | 0.038* | 0.133 | 0.208 |
| Bark effect group | Mass loss percentage | K | 0.091 | 0.135 | 0.091 | 0.332 |
| Bark effect group | Mass loss percentage | Ca | 0.217 | 0.002* | 0.217 | 0.048 * |
| Bark effect group | Mass loss percentage | Fe | 0.123 | 0.068 | 0.123 | 0.237 |
| Bark effect group | Mass loss percentage | Mn | 0.139 | 0.028* | 0.139 | 0.207 |
| Bark effect group | Mass loss percentage | Cu | 0.132 | 0.035* | 0.132 | 0.208 |
| Bark effect group | Mass loss percentage | Zn | 0.132 | 0.057* | 0.132 | 0.231 |
| Bark effect group | Mass loss percentage | Lignin | 0.141 | 0.026* | 0.141 | 0.207 |
| Bark effect group | Mass loss percentage | Sugar | 0.002 | 0.442 | 0.002 | 0.610 |
| Bark effect group | Mass loss percentage | Tannins | -0.036 | 0.666 | -0.036 | 0.794 |
| Bark effect group | Mass loss percentage | Fiber content | 0.175 | 0.006* | 0.175 | 0.072 |
| Bark effect group | Mass loss percentage | Hemicellulose | -0.028 | 0.606 | -0.028 | 0.754 |
| Bark effect group | Mass loss percentage | Cellulose | 0.114 | 0.054* | 0.114 | 0.230 |
| Bark effect group | Mass loss percentage | Water content | 0.142 | 0.049* | 0.142 | 0.228 |
| Bark effect group | Total (fungal and bacterial) biomass | C | 0.068 | 0.152 | 0.068 | 0.347 |
| Bark effect group | Total (fungal and bacterial) biomass | N | 0.083 | 0.106 | 0.083 | 0.304 |
| Bark effect group | Total (fungal and bacterial) biomass | P | 0.059 | 0.181 | 0.059 | 0.371 |
| Bark effect group | Total (fungal and bacterial) biomass | K | 0.141 | 0.051* | 0.141 | 0.228 |
| Bark effect group | Total (fungal and bacterial) biomass | Ca | 0.080 | 0.127 | 0.080 | 0.330 |
| Bark effect group | Total (fungal and bacterial) biomass | Fe | 0.101 | 0.086 | 0.101 | 0.271 |
| Bark effect group | Total (fungal and bacterial) biomass | Mn | 0.017 | 0.344 | 0.017 | 0.543 |
| Bark effect group | Total (fungal and bacterial) biomass | Cu | 0.028 | 0.30 | 0.028 | 0.501 |
| Bark effect group | Total (fungal and bacterial) biomass | Zn | 0.070 | 0.173 | 0.070 | 0.371 |
| Bark effect group | Total (fungal and bacterial) biomass | Lignin | 0.017 | 0.376 | 0.017 | 0.563 |
| Bark effect group | Total (fungal and bacterial) biomass | Sugar | 0.136 | 0.046* | 0.137 | 0.226 |
| Bark effect group | Total (fungal and bacterial) biomass | Tannins | 0.022 | 0.367 | 0.022 | 0.555 |
| Bark effect group | Total (fungal and bacterial) biomass | Fiber content | 0.004 | 0.416 | 0.004 | 0.596 |
| Bark effect group | Total (fungal and bacterial) biomass | Hemicellulose | 0.002 | 0.452 | 0.002 | 0.610 |
| Bark effect group | Total (fungal and bacterial) biomass | Cellulose | 0.025 | 0.296 | 0.025 | 0.499 |
| Bark effect group | Total (fungal and bacterial) biomass | Water content | 0.074 | 0.147 | 0.074 | 0.344 |
| Bark effect group | Fungal biomass | C | 0.398 | 0.001* | 0.398 | 0.048 * |
| Bark effect group | Fungal biomass | N | 0.331 | 0.002* | 0.331 | 0.048 * |
| Bark effect group | Fungal biomass | P | 0.346 | 0.002* | 0.346 | 0.048 * |
| Bark effect group | Fungal biomass | K | 0.464 | 0.002* | 0.464 | 0.048 * |
| Bark effect group | Fungal biomass | Ca | 0.446 | 0.001* | 0.446 | 0.048 * |
| Bark effect group | Fungal biomass | Fe | 0.278 | 0.005* | 0.278 | 0.069 |
| Bark effect group | Fungal biomass | Mn | 0.170 | 0.036* | 0.170 | 0.208 |
| Bark effect group | Fungal biomass | Cu | 0.195 | 0.016* | 0.195 | 0.154 |
| Bark effect group | Fungal biomass | Zn | 0.391 | 0.004* | 0.391 | 0.069 |
| Bark effect group | Fungal biomass | Lignin | 0.106 | 0.103 | 0.106 | 0.300 |
| Bark effect group | Fungal biomass | Sugar | -0.085 | 0.801 | -0.085 | 0.874 |
| Bark effect group | Fungal biomass | Tannins | -0.071 | 0.733 | -0.071 | 0.833 |
| Bark effect group | Fungal biomass | Fiber content | -0.029 | 0.660 | -0.029 | 0.792 |
| Bark effect group | Fungal biomass | Hemicellulose | 0.263 | 0.009* | 0.263 | 0.102 |
| Bark effect group | Fungal biomass | Cellulose | 0.007 | 0.451 | 0.007 | 0.610 |
| Bark effect group | Fungal biomass | Water content | 0.082 | 0.185 | 0.082 | 0.371 |
| Bark effect group | Bacterial biomass | C | 0.259 | 0.006* | 0.259 | 0.072 |
| Bark effect group | Bacterial biomass | N | 0.223 | 0.004* | 0.223 | 0.069 |
| Bark effect group | Bacterial biomass | P | 0.249 | 0.005* | 0.249 | 0.069 |
| Bark effect group | Bacterial biomass | K | 0.292 | 0.005* | 0.292 | 0.069 |
| Bark effect group | Bacterial biomass | Ca | 0.367 | 0.001* | 0.367 | 0.048 * |
| Bark effect group | Bacterial biomass | Fe | 0.093 | 0.135 | 0.093 | 0.332 |
| Bark effect group | Bacterial biomass | Mn | 0.104 | 0.092 | 0.104 | 0.285 |
| Bark effect group | Bacterial biomass | Cu | 0.122 | 0.062 | 0.122 | 0.231 |
| Bark effect group | Bacterial biomass | Zn | 0.229 | 0.015* | 0.229 | 0.152 |
| Bark effect group | Bacterial biomass | Lignin | 0.140 | 0.049* | 0.140 | 0.228 |
| Bark effect group | Bacterial biomass | Sugar | -0.032 | 0.592 | -0.032 | 0.744 |
| Bark effect group | Bacterial biomass | Tannins | -0.027 | 0.593 | -0.027 | 0.744 |
| Bark effect group | Bacterial biomass | Fiber content | 0.015 | 0.394 | 0.015 | 0.573 |
| Bark effect group | Bacterial biomass | Hemicellulose | 0.076 | 0.192 | 0.076 | 0.371 |
| Bark effect group | Bacterial biomass | Cellulose | -0.066 | 0.780 | -0.066 | 0.871 |
| Bark effect group | Bacterial biomass | Water content | 0.157 | 0.071 | 0.157 | 0.243 |
| Non-bark effect group | Ascomycota | C | 0.138 | 0.149 | 0.138 | 0.311 |
| Non-bark effect group | Ascomycota | N | 0.256 | 0.049* | 0.256 | 0.196 |
| Non-bark effect group | Ascomycota | P | 0.221 | 0.075 | 0.221 | 0.221 |
| Non-bark effect group | Ascomycota | K | -0.005 | 0.452 | -0.005 | 0.643 |
| Non-bark effect group | Ascomycota | Ca | 0.191 | 0.087 | 0.191 | 0.235 |
| Non-bark effect group | Ascomycota | Fe | -0.082 | 0.801 | -0.082 | 0.903 |
| Non-bark effect group | Ascomycota | Mn | -0.007 | 0.433 | -0.007 | 0.630 |
| Non-bark effect group | Ascomycota | Cu | 0.191 | 0.069 | 0.191 | 0.219 |
| Non-bark effect group | Ascomycota | Zn | 0.033 | 0.290 | 0.033 | 0.467 |
| Non-bark effect group | Ascomycota | Lignin | 0.126 | 0.154 | 0.126 | 0.318 |
| Non-bark effect group | Ascomycota | Sugar | 0.074 | 0.189 | 0.074 | 0.363 |
| Non-bark effect group | Ascomycota | Tannins | 0.072 | 0.206 | 0.072 | 0.384 |
| Non-bark effect group | Ascomycota | Fiber content | 0.288 | 0.026* | 0.288 | 0.161 |
| Non-bark effect group | Ascomycota | Hemicellulose | 0.326 | 0.012* | 0.326 | 0.139 |
| Non-bark effect group | Ascomycota | Cellulose | 0.357 | 0.021* | 0.357 | 0.146 |
| Non-bark effect group | Ascomycota | Water content | -0.077 | 0.651 | -0.077 | 0.801 |
| Non-bark effect group | Basidiomycota | C | 0.159 | 0.139 | 0.159 | 0.304 |
| Non-bark effect group | Basidiomycota | N | 0.270 | 0.034* | 0.270 | 0.181 |
| Non-bark effect group | Basidiomycota | P | 0.250 | 0.068 | 0.250 | 0.219 |
| Non-bark effect group | Basidiomycota | K | 0.002 | 0.439 | 0.002 | 0.634 |
| Non-bark effect group | Basidiomycota | Ca | 0.206 | 0.085 | 0.206 | 0.235 |
| Non-bark effect group | Basidiomycota | Fe | -0.070 | 0.741 | -0.070 | 0.884 |
| Non-bark effect group | Basidiomycota | Mn | 0.001 | 0.417 | 0.001 | 0.621 |
| Non-bark effect group | Basidiomycota | Cu | 0.216 | 0.061 | 0.216 | 0.213 |
| Non-bark effect group | Basidiomycota | Zn | 0.054 | 0.242 | 0.054 | 0.415 |
| Non-bark effect group | Basidiomycota | Lignin | 0.146 | 0.115 | 0.146 | 0.273 |
| Non-bark effect group | Basidiomycota | Sugar | 0.089 | 0.177 | 0.089 | 0.347 |
| Non-bark effect group | Basidiomycota | Tannins | 0.093 | 0.146 | 0.093 | 0.308 |
| Non-bark effect group | Basidiomycota | Fiber content | 0.312 | 0.032* | 0.312 | 0.181 |
| Non-bark effect group | Basidiomycota | Hemicellulose | 0.360 | 0.013* | 0.360 | 0.139 |
| Non-bark effect group | Basidiomycota | Cellulose | 0.370 | 0.020* | 0.370 | 0.146 |
| Non-bark effect group | Basidiomycota | Water content | -0.106 | 0.769 | -0.106 | 0.892 |
| Non-bark effect group | Saprotroph | C | 0.206 | 0.056 | 0.206 | 0.210 |
| Non-bark effect group | Saprotroph | N | 0.251 | 0.037* | 0.251 | 0.187 |
| Non-bark effect group | Saprotroph | P | 0.221 | 0.058 | 0.221 | 0.210 |
| Non-bark effect group | Saprotroph | K | 0.066 | 0.268 | 0.066 | 0.440 |
| Non-bark effect group | Saprotroph | Ca | 0.166 | 0.112 | 0.166 | 0.273 |
| Non-bark effect group | Saprotroph | Fe | 0.075 | 0.193 | 0.075 | 0.367 |
| Non-bark effect group | Saprotroph | Mn | 0.188 | 0.061 | 0.188 | 0.213 |
| Non-bark effect group | Saprotroph | Cu | 0.228 | 0.040* | 0.228 | 0.187 |
| Non-bark effect group | Saprotroph | Zn | 0.133 | 0.092 | 0.133 | 0.236 |
| Non-bark effect group | Saprotroph | Lignin | 0.223 | 0.043* | 0.223 | 0.192 |
| Non-bark effect group | Saprotroph | Sugar | 0.057 | 0.253 | 0.057 | 0.419 |
| Non-bark effect group | Saprotroph | Tannins | 0.139 | 0.092 | 0.139 | 0.236 |
| Non-bark effect group | Saprotroph | Fiber content | 0.329 | 0.009* | 0.329 | 0.139 |
| Non-bark effect group | Saprotroph | Hemicellulose | 0.340 | 0.006* | 0.340 | 0.139 |
| Non-bark effect group | Saprotroph | Cellulose | 0.374 | 0.004* | 0.374 | 0.110 |
| Non-bark effect group | Saprotroph | Water content | -0.035 | 0.551 | -0.035 | 0.740 |
| Non-bark effect group | Plant pathogen | C | 0.031 | 0.344 | 0.031 | 0.537 |
| Non-bark effect group | Plant pathogen | N | -0.084 | 0.616 | -0.084 | 0.794 |
| Non-bark effect group | Plant pathogen | P | 0.143 | 0.238 | 0.143 | 0.415 |
| Non-bark effect group | Plant pathogen | K | 0.137 | 0.226 | 0.137 | 0.402 |
| Non-bark effect group | Plant pathogen | Ca | 0.075 | 0.294 | 0.075 | 0.467 |
| Non-bark effect group | Plant pathogen | Fe | 0.178 | 0.072 | 0.178 | 0.219 |
| Non-bark effect group | Plant pathogen | Mn | 0.138 | 0.173 | 0.138 | 0.342 |
| Non-bark effect group | Plant pathogen | Cu | 0.161 | 0.179 | 0.161 | 0.347 |
| Non-bark effect group | Plant pathogen | Zn | 0.150 | 0.121 | 0.150 | 0.277 |
| Non-bark effect group | Plant pathogen | Lignin | 0.427 | 0.017* | 0.427 | 0.146 |
| Non-bark effect group | Plant pathogen | Sugar | 0.205 | 0.081 | 0.205 | 0.232 |
| Non-bark effect group | Plant pathogen | Tannins | 0.104 | 0.202 | 0.104 | 0.380 |
| Non-bark effect group | Plant pathogen | Fiber content | 0.256 | 0.085 | 0.256 | 0.235 |
| Non-bark effect group | Plant pathogen | Hemicellulose | -0.045 | 0.585 | -0.045 | 0.764 |
| Non-bark effect group | Plant pathogen | Cellulose | 0.161 | 0.216 | 0.161 | 0.395 |
| Non-bark effect group | Plant pathogen | Water content | 0.029 | 0.373 | 0.029 | 0.564 |
| Non-bark effect group | Ectomycorrhizal | C | -0.229 | 0.986 | -0.229 | 0.989 |
| Non-bark effect group | Ectomycorrhizal | N | -0.171 | 0.833 | -0.171 | 0.905 |
| Non-bark effect group | Ectomycorrhizal | P | -0.242 | 0.939 | -0.242 | 0.975 |
| Non-bark effect group | Ectomycorrhizal | K | -0.173 | 0.826 | -0.173 | 0.905 |
| Non-bark effect group | Ectomycorrhizal | Ca | -0.124 | 0.729 | -0.124 | 0.875 |
| Non-bark effect group | Ectomycorrhizal | Fe | -0.159 | 0.964 | -0.159 | 0.989 |
| Non-bark effect group | Ectomycorrhizal | Mn | -0.111 | 0.767 | -0.111 | 0.892 |
| Non-bark effect group | Ectomycorrhizal | Cu | -0.118 | 0.722 | -0.118 | 0.872 |
| Non-bark effect group | Ectomycorrhizal | Zn | -0.198 | 0.988 | -0.198 | 0.989 |
| Non-bark effect group | Ectomycorrhizal | Lignin | -0.157 | 0.785 | -0.157 | 0.901 |
| Non-bark effect group | Ectomycorrhizal | Sugar | -0.131 | 0.831 | -0.131 | 0.905 |
| Non-bark effect group | Ectomycorrhizal | Tannins | 0.001 | 0.429 | 0.001 | 0.629 |
| Non-bark effect group | Ectomycorrhizal | Fiber content | -0.152 | 0.788 | -0.152 | 0.901 |
| Non-bark effect group | Ectomycorrhizal | Hemicellulose | -0.156 | 0.864 | -0.156 | 0.911 |
| Non-bark effect group | Ectomycorrhizal | Cellulose | -0.176 | 0.804 | -0.176 | 0.903 |
| Non-bark effect group | Ectomycorrhizal | Water content | -0.021 | 0.451 | -0.021 | 0.643 |
| Non-bark effect group | Soft rot | C | -0.132 | 0.771 | -0.132 | 0.892 |
| Non-bark effect group | Soft rot | N | -0.102 | 0.635 | -0.102 | 0.800 |
| Non-bark effect group | Soft rot | P | -0.053 | 0.555 | -0.053 | 0.740 |
| Non-bark effect group | Soft rot | K | -0.039 | 0.535 | -0.039 | 0.729 |
| Non-bark effect group | Soft rot | Ca | -0.176 | 0.848 | -0.176 | 0.905 |
| Non-bark effect group | Soft rot | Fe | -0.014 | 0.509 | -0.014 | 0.703 |
| Non-bark effect group | Soft rot | Mn | -0.208 | 0.979 | -0.208 | 0.989 |
| Non-bark effect group | Soft rot | Cu | -0.146 | 0.793 | -0.146 | 0.901 |
| Non-bark effect group | Soft rot | Zn | -0.056 | 0.609 | -0.056 | 0.790 |
| Non-bark effect group | Soft rot | Lignin | -0.106 | 0.672 | -0.106 | 0.822 |
| Non-bark effect group | Soft rot | Sugar | -0.149 | 0.861 | -0.149 | 0.911 |
| Non-bark effect group | Soft rot | Tannins | -0.131 | 0.896 | -0.131 | 0.935 |
| Non-bark effect group | Soft rot | Fiber content | -0.168 | 0.822 | -0.168 | 0.905 |
| Non-bark effect group | Soft rot | Hemicellulose | -0.140 | 0.809 | -0.140 | 0.903 |
| Non-bark effect group | Soft rot | Cellulose | -0.201 | 0.879 | -0.201 | 0.922 |
| Non-bark effect group | Soft rot | Water content | -0.064 | 0.575 | -0.064 | 0.756 |
| Non-bark effect group | White rot | C | 0.106 | 0.226 | 0.106 | 0.402 |
| Non-bark effect group | White rot | N | -0.054 | 0.561 | -0.054 | 0.743 |
| Non-bark effect group | White rot | P | 0.228 | 0.134 | 0.228 | 0.303 |
| Non-bark effect group | White rot | K | 0.230 | 0.101 | 0.230 | 0.255 |
| Non-bark effect group | White rot | Ca | 0.187 | 0.164 | 0.187 | 0.335 |
| Non-bark effect group | White rot | Fe | 0.270 | 0.022* | 0.270 | 0.146 |
| Non-bark effect group | White rot | Mn | 0.426 | 0.002* | 0.426 | 0.096 |
| Non-bark effect group | White rot | Cu | 0.256 | 0.072 | 0.256 | 0.219 |
| Non-bark effect group | White rot | Zn | 0.154 | 0.113 | 0.154 | 0.273 |
| Non-bark effect group | White rot | Lignin | 0.439 | 0.021* | 0.439 | 0.146 |
| Non-bark effect group | White rot | Sugar | 0.349 | 0.018 | 0.349 | 0.146 |
| Non-bark effect group | White rot | Tannins | 0.133 | 0.141 | 0.133 | 0.304 |
| Non-bark effect group | White rot | Fiber content | 0.282 | 0.076 | 0.282 | 0.221 |
| Non-bark effect group | White rot | Hemicellulose | 0.038 | 0.390 | 0.038 | 0.585 |
| Non-bark effect group | White rot | Cellulose | 0.171 | 0.171 | 0.171 | 0.342 |
| Non-bark effect group | White rot | Water content | 0.305 | 0.046* | 0.305 | 0.192 |
| Non-bark effect group | Brown rot | C | -0.208 | 0.974 | -0.208 | 0.989 |
| Non-bark effect group | Brown rot | N | 0.003 | 0.428 | 0.003 | 0.629 |
| Non-bark effect group | Brown rot | P | -0.172 | 0.764 | -0.172 | 0.892 |
| Non-bark effect group | Brown rot | K | -0.170 | 0.763 | -0.170 | 0.892 |
| Non-bark effect group | Brown rot | Ca | -0.026 | 0.471 | -0.026 | 0.665 |
| Non-bark effect group | Brown rot | Fe | -0.179 | 0.989 | -0.179 | 0.989 |
| Non-bark effect group | Brown rot | Mn | -0.160 | 0.846 | -0.160 | 0.905 |
| Non-bark effect group | Brown rot | Cu | -0.093 | 0.634 | -0.093 | 0.800 |
| Non-bark effect group | Brown rot | Zn | -0.203 | 0.979 | -0.203 | 0.989 |
| Non-bark effect group | Brown rot | Lignin | -0.107 | 0.638 | -0.107 | 0.800 |
| Non-bark effect group | Brown rot | Sugar | -0.137 | 0.841 | -0.137 | 0.905 |
| Non-bark effect group | Brown rot | Tannins | -0.065 | 0.693 | -0.065 | 0.842 |
| Non-bark effect group | Brown rot | Fiber content | -0.089 | 0.649 | -0.089 | 0.801 |
| Non-bark effect group | Brown rot | Hemicellulose | -0.159 | 0.847 | -0.159 | 0.905 |
| Non-bark effect group | Brown rot | Cellulose | -0.047 | 0.544 | -0.047 | 0.736 |
| Non-bark effect group | Brown rot | Water content | 0.178 | 0.171 | 0.178 | 0.342 |
| Non-bark effect group | Mass loss percentage | C | 0.084 | 0.276 | 0.084 | 0.449 |
| Non-bark effect group | Mass loss percentage | N | -0.071 | 0.620 | -0.071 | 0.794 |
| Non-bark effect group | Mass loss percentage | P | 0.271 | 0.087 | 0.271 | 0.235 |
| Non-bark effect group | Mass loss percentage | K | 0.357 | 0.036* | 0.357 | 0.187 |
| Non-bark effect group | Mass loss percentage | Ca | 0.106 | 0.253 | 0.106 | 0.419 |
| Non-bark effect group | Mass loss percentage | Fe | 0.236 | 0.045* | 0.236 | 0.192 |
| Non-bark effect group | Mass loss percentage | Mn | 0.293 | 0.033* | 0.293 | 0.181 |
| Non-bark effect group | Mass loss percentage | Cu | 0.290 | 0.052 | 0.290 | 0.204 |
| Non-bark effect group | Mass loss percentage | Zn | 0.210 | 0.066 | 0.210 | 0.219 |
| Non-bark effect group | Mass loss percentage | Lignin | 0.517 | 0.009* | 0.517 | 0.139 |
| Non-bark effect group | Mass loss percentage | Sugar | 0.264 | 0.040* | 0.264 | 0.187 |
| Non-bark effect group | Mass loss percentage | Tannins | 0.146 | 0.105 | 0.146 | 0.262 |
| Non-bark effect group | Mass loss percentage | Fiber content | 0.330 | 0.031* | 0.330 | 0.181 |
| Non-bark effect group | Mass loss percentage | Hemicellulose | 0.034 | 0.370 | 0.034 | 0.564 |
| Non-bark effect group | Mass loss percentage | Cellulose | 0.229 | 0.114 | 0.229 | 0.273 |
| Non-bark effect group | Mass loss percentage | Water content | 0.251 | 0.070 | 0.251 | 0.219 |
| Non-bark effect group | Total (fungal and bacterial) biomass | C | 0.193 | 0.138 | 0.193 | 0.304 |
| Non-bark effect group | Total (fungal and bacterial) biomass | N | -0.096 | 0.642 | -0.096 | 0.800 |
| Non-bark effect group | Total (fungal and bacterial) biomass | P | 0.153 | 0.218 | 0.153 | 0.395 |
| Non-bark effect group | Total (fungal and bacterial) biomass | K | 0.330 | 0.057* | 0.330 | 0.210 |
| Non-bark effect group | Total (fungal and bacterial) biomass | Ca | 0.090 | 0.250 | 0.090 | 0.419 |
| Non-bark effect group | Total (fungal and bacterial) biomass | Fe | 0.288 | 0.019* | 0.288 | 0.146 |
| Non-bark effect group | Total (fungal and bacterial) biomass | Mn | 0.162 | 0.141 | 0.162 | 0.304 |
| Non-bark effect group | Total (fungal and bacterial) biomass | Cu | 0.193 | 0.120 | 0.193 | 0.277 |
| Non-bark effect group | Total (fungal and bacterial) biomass | Zn | 0.293 | 0.011* | 0.293 | 0.139 |
| Non-bark effect group | Total (fungal and bacterial) biomass | Lignin | 0.342 | 0.034* | 0.342 | 0.181 |
| Non-bark effect group | Total (fungal and bacterial) biomass | Sugar | 0.182 | 0.117 | 0.182 | 0.274 |
| Non-bark effect group | Total (fungal and bacterial) biomass | Tannins | 0.164 | 0.092 | 0.164 | 0.236 |
| Non-bark effect group | Total (fungal and bacterial) biomass | Fiber content | 0.198 | 0.145 | 0.198 | 0.308 |
| Non-bark effect group | Total (fungal and bacterial) biomass | Hemicellulose | 0.063 | 0.308 | 0.063 | 0.485 |
| Non-bark effect group | Total (fungal and bacterial) biomass | Cellulose | 0.134 | 0.233 | 0.134 | 0.410 |
| Non-bark effect group | Total (fungal and bacterial) biomass | Water content | 0.079 | 0.294 | 0.079 | 0.467 |
| Non-bark effect group | Fungal biomass | C | 0.471 | 0.001* | 0.471 | 0.096 |
| Non-bark effect group | Fungal biomass | N | 0.349 | 0.018* | 0.349 | 0.146 |
| Non-bark effect group | Fungal biomass | P | 0.357 | 0.018* | 0.357 | 0.146 |
| Non-bark effect group | Fungal biomass | K | 0.115 | 0.214 | 0.115 | 0.395 |
| Non-bark effect group | Fungal biomass | Ca | 0.242 | 0.064* | 0.242 | 0.219 |
| Non-bark effect group | Fungal biomass | Fe | 0.186 | 0.056* | 0.186 | 0.210 |
| Non-bark effect group | Fungal biomass | Mn | -0.015 | 0.486 | -0.015 | 0.676 |
| Non-bark effect group | Fungal biomass | Cu | 0.269 | 0.039* | 0.269 | 0.187 |
| Non-bark effect group | Fungal biomass | Zn | 0.303 | 0.010* | 0.303 | 0.139 |
| Non-bark effect group | Fungal biomass | Lignin | 0.214 | 0.075 | 0.214 | 0.221 |
| Non-bark effect group | Fungal biomass | Sugar | 0.303 | 0.023* | 0.303 | 0.147 |
| Non-bark effect group | Fungal biomass | Tannins | 0.208 | 0.044* | 0.208 | 0.192 |
| Non-bark effect group | Fungal biomass | Fiber content | 0.386 | 0.012* | 0.386 | 0.139 |
| Non-bark effect group | Fungal biomass | Hemicellulose | 0.533 | 0.001* | 0.533 | 0.096 |
| Non-bark effect group | Fungal biomass | Cellulose | 0.395 | 0.013* | 0.395 | 0.139 |
| Non-bark effect group | Fungal biomass | Water content | 0.079 | 0.242 | 0.079 | 0.415 |
| Non-bark effect group | Bacterial biomass | C | 0.030 | 0.355 | 0.030 | 0.547 |
| Non-bark effect group | Bacterial biomass | N | 0.339 | 0.009* | 0.339 | 0.139 |
| Non-bark effect group | Bacterial biomass | P | 0.194 | 0.071 | 0.194 | 0.219 |
| Non-bark effect group | Bacterial biomass | K | 0.061 | 0.251 | 0.061 | 0.419 |
| Non-bark effect group | Bacterial biomass | Ca | 0.431 | 0.002* | 0.431 | 0.096 |
| Non-bark effect group | Bacterial biomass | Fe | -0.021 | 0.519 | -0.021 | 0.712 |
| Non-bark effect group | Bacterial biomass | Mn | 0.027 | 0.356 | 0.027 | 0.547 |
| Non-bark effect group | Bacterial biomass | Cu | 0.260 | 0.022* | 0.260 | 0.146 |
| Non-bark effect group | Bacterial biomass | Zn | -0.011 | 0.476 | -0.011 | 0.667 |
| Non-bark effect group | Bacterial biomass | Lignin | 0.358 | 0.008* | 0.358 | 0.139 |
| Non-bark effect group | Bacterial biomass | Sugar | 0.194 | 0.046* | 0.194 | 0.192 |
| Non-bark effect group | Bacterial biomass | Tannins | 0.196 | 0.047* | 0.196 | 0.192 |
| Non-bark effect group | Bacterial biomass | Fiber content | 0.397 | 0.004* | 0.397 | 0.110 |
| Non-bark effect group | Bacterial biomass | Hemicellulose | 0.150 | 0.092 | 0.150 | 0.236 |
| Non-bark effect group | Bacterial biomass | Cellulose | 0.438 | 0.003* | 0.438 | 0.110 |
| Non-bark effect group | Bacterial biomass | Water content | 0.323 | 0.018* | 0.323 | 0.146 |
